# Predicting the distribution of *Ixodes ricinus* and *Dermacentor reticulatus* in Europe: a comparison of climate niche modelling approaches

**DOI:** 10.1186/s13071-023-05959-y

**Published:** 2023-10-25

**Authors:** Madeleine Noll, Richard Wall, Benjamin L. Makepeace, Hannah Newbury, Lukasz Adaszek, René Bødker, Agustín Estrada-Peña, Jacques Guillot, Isabel Pereira da Fonseca, Julia Probst, Paul Overgaauw, Christina Strube, Fathiah Zakham, Stefania Zanet, Hannah Rose Vineer

**Affiliations:** 1 Institute of Infection, Veterinary and Ecological Sciences, University of Liverpool, Liverpool, UK; 2 School of Biological Sciences, University of Bristol, Bristol, UK; 3 MSD Animal Health, Walton, Milton Keynes, UK; 4 Department of Epizootiology and Clinic of Infectious Diseases, Faculty of Veterinary Medicine, University of Life Sciences, Lublin, Poland; 5 Section of Animal Welfare and Disease Control, Department of Veterinary and Animal Sciences, University of Copenhagen, Copenhagen, Denmark; 6 Department of Animal Health, Faculty of Veterinary Medicine, University of Zaragoza, Saragossa, Spain; 7 Instituto Agroalimentario de Aragón (IA2), Saragossa, Spain; 8 Department of Dermatology-Parasitology-Mycology, École Nationale Vétérinaire, Oniris, Nantes, France; 9 CIISA-Centre for Interdisciplinary Research in Animal Health, Faculty of Veterinary Medicine, University of Lisbon, Lisbon, Portugal; 10 Associate Laboratory for Animal and Veterinary Sciences (AL4AnimalS), Vila Real, Portugal; 11 Institute for Parasitology, Centre for Infection Medicine, University of Veterinary Medicine Hannover, Hannover, Germany; 12 Department Population Health Sciences, Division of Veterinary Public Health, Faculty of Veterinary Medicine, Institute for Risk Assessment Sciences, Utrecht University, Utrecht, The Netherlands; 13 Department of Virology, Faculty of Medicine, University of Helsinki, Helsinki, Finland; 14 Department of Veterinary Biosciences, Faculty of Veterinary Medicine, University of Helsinki, Helsinki, Finland; 15 Department of Veterinary Sciences, University of Turin, Grugliasco, Italy

**Keywords:** Species distribution modelling, Climate niche modelling, Ticks, Climate niche, Climate change, *Ixodes*, *Dermacentor*

## Abstract

**Background:**

The ticks *Ixodes ricinus* and *Dermacentor reticulatus* are two of the most important vectors in Europe. Climate niche modelling has been used in many studies to attempt to explain their distribution and to predict changes under a range of climate change scenarios. The aim of this study was to assess the ability of different climate niche modelling approaches to explain the known distribution of *I. ricinus* and *D. reticulatus* in Europe.

**Methods:**

A series of climate niche models, using different combinations of input data, were constructed and assessed. Species occurrence records obtained from systematic literature searches and Global Biodiversity Information Facility data were thinned to different degrees to remove sampling spatial bias. Four sources of climate data were used: bioclimatic variables, WorldClim, TerraClimate and MODIS satellite-derived data. Eight different model training extents were examined and three modelling frameworks were used: maximum entropy, generalised additive models and random forest models. The results were validated through internal cross-validation, comparison with an external independent dataset and expert opinion.

**Results:**

The performance metrics and predictive ability of the different modelling approaches varied significantly within and between each species. Different combinations were better able to define the distribution of each of the two species. However, no single approach was considered fully able to capture the known distribution of the species. When considering the mean of the performance metrics of internal and external validation, 24 models for *I. ricinus* and 11 models for *D. reticulatus* of the 96 constructed were considered adequate according to the following criteria: area under the receiver-operating characteristic curve > 0.7; true skill statistic > 0.4; Miller’s calibration slope 0.25 above or below 1; Boyce index > 0.9; omission rate < 0.15.

**Conclusions:**

This comprehensive analysis suggests that there is no single ‘best practice’ climate modelling approach to account for the distribution of these tick species. This has important implications for attempts to predict climate-mediated impacts on future tick distribution. It is suggested here that climate variables alone are not sufficient; habitat type, host availability and anthropogenic impacts, not included in current modelling approaches, could contribute to determining tick presence or absence at the local or regional scale.

**Graphical abstract:**

**Supplementary Information:**

The online version contains supplementary material available at 10.1186/s13071-023-05959-y.

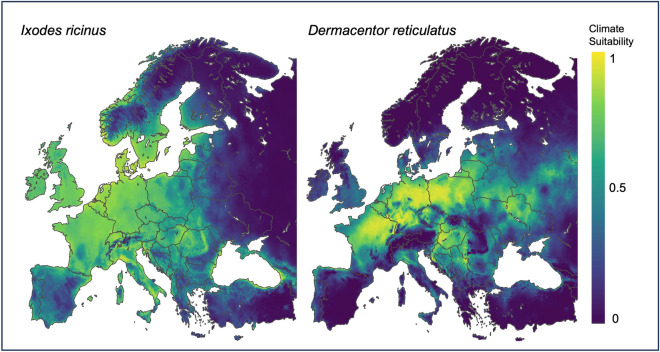

## Introduction

Ticks are obligate hematophagous arthropods of global importance because of their public and veterinary health impacts [1]. *Ixodes ricinus* is the most widespread tick in Europe, with its distribution extending across much of the continent [2]. It is considered to be of serious health concern due to its extensive range of vertebrate hosts and ability to transmit a variety of pathogens, including the causative agents of several important zoonotic diseases such as Lyme borreliosis, tick-borne encephalitis and anaplasmosis [3, 4]. The second most-reported tick in Europe is *Dermacentor reticulatus*, and although there is overlap in the ranges of *I. ricinus* and *D. reticulatus*, the latter has a narrower distribution based in central Europe [5]. *Dermacentor reticulatus* is a vector of causal agents of diseases, such as canine babesiosis and equine piroplasmosis [6]. As a result of the risks these tick species pose to humans, livestock and companion animals, understanding their distributions and how these may change in future are issues of research importance.

As generalist parasites that spend most of their life cycle off their hosts, these three-host ticks are particularly sensitive to temperature and humidity, since these affect the rates of physiological activity and desiccation, and this determines their ability to quest and survive [7, 8]. *Ixodes ricinus* requires an environment where the relative humidity is > 80% [7], and the mean daily air temperature exceeds 5 °C for approximately 170 days a year [9]; therefore, the species extends from Scandinavia to the Mediterranean basin in Europe [2]. *Dermacentor reticulatus* is more cold-tolerant than *I. ricinus* [7, 10] and may be active throughout winter [10].

The role of climate in determining tick behaviour, survival and distribution has made them popular subjects for species distribution modelling. Species distribution models (SDMs), also known as ecological and climate niche models, are potentially powerful research tools that can be used to estimate the suitability of a region for a species in time and space [11]. Species distribution models work on the assumption that species are in equilibrium with their environment and hence aim to define the environmental parameters of the species’ niche by finding statistical associations between key environmental variables and the presence and absence of a species in that location [12, 13]. Once the species’ niche has been captured, it can be projected onto different spatial or temporal spaces to predict the suitability of different environments for the species of interest. Although the resultant maps are often referred to as the predicted distribution of the species, they represent a measure of statistical similarity between the environmental variables in each grid cell, or pixel, and the niche of the species derived from the input data [13]. Due to their simplicity and rapidity, these models have been widely integrated into ecological studies mapping the distribution of vectors, including ticks [11], particularly under different climate change scenarios [14–16].

However, SDM output is heavily influenced by the modelling algorithm and parameters [17], the size of the region used to train the model [18], species data [19] and the ability of the environmental variables to fully capture the niche of the species [20]. As a result, studies that have attempted to explore the distribution of ticks in Europe, and their future climate-mediated changes, differ widely in the areas predicted as environmentally suitable [14–16]. It is important to determine which modelling approach best captures the niche of the species to effectively predict environmental suitability across Europe. This is particularly significant if the niche is going to be projected to estimate future suitability. In a medical and veterinary context, and given the clinical risk, confidence in the reliability of SDM outputs is important if the maps are to be used as a proxy for the distribution of the species.

This study aimed to compare multiple modelling approaches in their ability to capture the niche of two important tick species in Europe, *I. ricinus* and *D. reticulatus,* to assess their potential usefulness in predicting climate suitability. Three modelling algorithms, four sets of explanatory variables and eight different training extents were used in different combinations to construct 96 modelling approaches. Each approach was subjected to internal and external statistical validation as well as a more subjective expert review.

## Methods

### Occurrence data

**Fig. 1:**
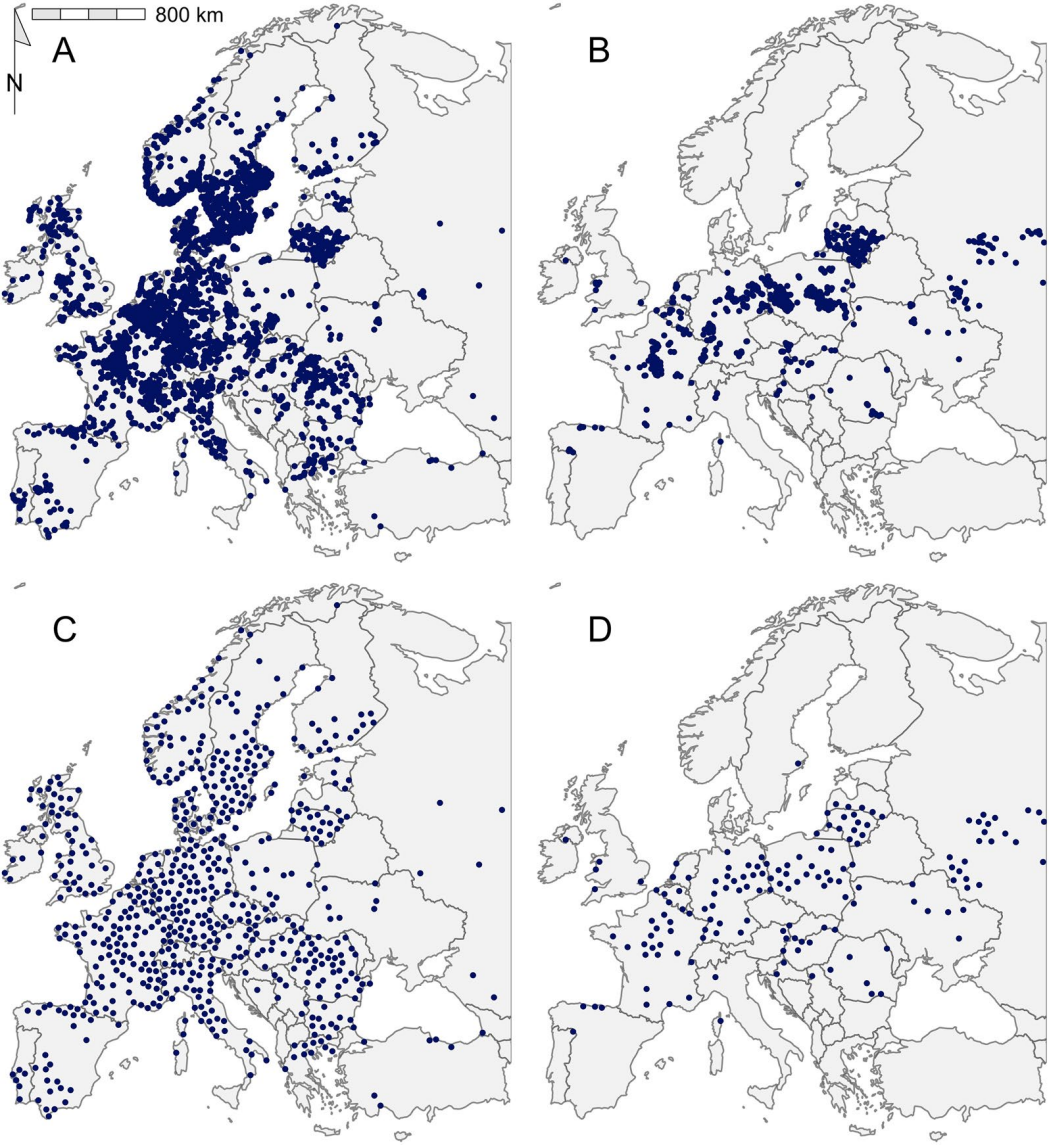
The georeferenced occurrence data for the complete *Ixodes ricinus* (**A**) and *Dermacentor reticulatus* dataset (**C**) and the spatially rarefied data used for model training for *I. ricinus* (**B**) and *D. reticulatus* (**D**). Points may overlap in panels (**A** and **C**)

Four sets of tick occurrence data were combined to build the models: data held by the Global Biodiversity Information Facility [21], data extracted from a systematic review of the literature published between 1970 and 2014 [22], a systematic review of the more recent literature between 2015 and 2021 [23] and additional publications which filled gaps in the previous sources. The additional publications were found through targeted searches for occurrence data in regions where tick distribution was not reflected in previous sources, for example the presence of *I. ricinus* in Portugal [24–27]. The combined occurrence dataset was then cleaned in sequential steps in R (v.4.2.1) [28, 29] to increase reliability and quality by removing entries which met the following criteria: (i) missing or errors in coordinates; (ii) duplicated; (iii) coordinates fell within 1000 m of country/province centroids, institutions or capital cities; (iv) coordinates outside of the area of interest (see Fig. 1 for area of interest) (Additional file 1: Dataset S1).

It is important for SDMs that the entire study area has been systematically or randomly sampled. To meet this assumption, the data were randomly spatially rarefied using several distance thresholds (10 km–100 km in 10-km increments). The spatial distribution of the resultant datasets was quantified using the nearest neighbour index (NNI) and the thinning distance threshold which resulted in a dataset with the NNI closest to 1, which represents a random distribution, was chosen for the occurrence data [30]. As true absence data for ticks are difficult to obtain, these presence data were then combined with 10,000 randomly generated pseudo-absence and background points for the use in presence-absence and presence only models, respectively [31–33]. The method of randomly selecting pseudo-absence and background points from across the training extent was chosen as it has the fewest assumptions and has proven to be adequate for different modelling algorithms [32, 33].

### Explanatory variables

Explanatory variables were obtained from four sources and included interpolated data and satellite imagery (Table 1). These variables were chosen as they had previously been used to capture the climatic niche of different tick species in Europe [16, 34, 35]. Monthly time series data, from WorldClim, TerraClimate and MODIS, were reduced by temporal Fourier transformation using an amended version of the R script provided by Estrada-Peña et al. [35, 36]. This technique reduces the time series data into annual harmonics (sine and cosine waves) with characteristic frequencies, amplitudes and phase angles which capture the annual behaviour of individual environmental variables including the start of spring and autumn as well as the duration of the summer [35]. This method is advantageous as it retains ecologically relevant information while being statistically sound and reducing the number of variables [35, 36].

**Table 1:** Data source of a range of explanatory variables used for the tick distribution modelling plus the reference for the published data source

Data source	Explanatory variable		Refs.
Bioclimatic variables (1970–2000)	Isothermality	(BIO3)	[65]
	Temperature seasonality	(BIO4)	
	Maximum temperature of warmest month	(BIO5)	
	Annual precipitation	(BIO12)	
	Precipitation of driest quarter	(BIO17)	
MODIS satellite *^a^ (2000–2020)	Normalized difference Vegetation Index	(MOD13X2 v061)	[57]
	Day land surface temperature/emissivity	(MOD11C3 v061)	
	Night land surface temperature/emissivity	(MOD11C3 v061)	
WorldClim *^a^ (1970–2020)	Average temperature	(AvgT)	[66]
	Precipitation	(Prec)	
TerraClimate *^b^ (1970–2021)	Maximum temperature	(MaxT)	[67]
	Minimum temperature	(MinT)	
	Water vapour deficit	(WVP)	
	Soil moisture	(SoilM)	

^*^Fourier transformed. Three^a^ or five^b^ coefficients from the Fourier transformation were used

All the variables were resampled to the same resolution (10 km × 10 km) and clipped to the study extent (Europe). Due to the pervasive influence of the training extent on model output, and as no consensus on the delimitation of this area has been made for ticks, several model training extents were tested by creating a 100 km, 200 km, 300 km, 400 km, 500 km, 600 km and 700 km buffer around the occurrence data for each species as well as using the whole study region [37, 38].

For the Fourier transformed datasets, the variables and numbers of coefficients used were based on previous studies defining the climatic niche of tick species [20, 34, 39]. However, different combinations of the 19 available bioclimatic variables have been used in previous studies; hence, a new selection was used here. To limit autocorrelation between bioclimatic variables, the variance inflation factor (VIF) of individual variables was assessed [40]. Due to the nature of climatic variables, it is impossible to eliminate correlation, but a VIF threshold of 10 can exclude the variables which would cause the greatest problems [40]. The collinearity was assessed over the whole extent of Europe to prevent a collinearity shift when projecting training extents [41]. However, one issue with the automated selection of variables is losing biologically relevant information. To avoid this, the VIF results were taken along with the ecological relevance of the variable, which was established through knowledge of the species’ ecology and principal component analysis (Additional files 2, 3). The final explanatory variables used in these models are shown in Table 1.

### Model implementation

#### Modelling methods

The choice of model can influence the predicted suitability for a species [17, 42]. Here, three SDM algorithms were assessed: generalised additive model (GAM) [43], random forest (RF) [44] and maximum entropy (MaxEnt) [45]. Individual model fitting and tuning were implemented for each algorithm. Default settings and equally weighted presence and pseudo-absence points were used for the GAM models implemented using the *mgcv* R package (v.1.8-38) [17, 32, 46]. Random forest models were run with 1000 trees and a down-sampling approach was employed, whereby each classification tree was made with equal presence and pseudo-absence data points, and pseudo-absences were randomly sampled with replacement from the training data [47]. RF models were implemented using the *randomForest* package in R (v4.6-14) [48]. The MaxEnt models were run with 10,000 background points and default parameters of the *dismo* R package (v.1.3-5) [49], as they have been shown to produce robust, well-performing models [50]. All models were trained with each set of explanatory variables and at each training extent referred to above and then projected to the whole of the Europe.

#### Validation

Spatial cross-validation was performed by geographically splitting the occurrence data into five systematically selected and assigned folds (300 km × 300 km), meaning that for each replication, 80% of the data was used for model training and the remaining 20% for model testing [51]. Therefore, for each combination of species, modelling algorithm, training extent and explanatory variable source, five SDMs were generated, resulting in 960 model outputs. Model performance was assessed using the discrimination metric, area under the receiver-operating characteristic curve (AUC), and the classification metric and true skill statistic (TSS), where performance thresholds were set at 0.7 and 0.4, respectively [52]. The goodness of calibration metrics, Miller’s calibration slope (MCS), Continuous Boyce Index (CBI) and omission rate (OR) were also used in model evaluation [53, 54]. The performance threshold for MCS was 0.25 above or below 1 and models with CBI values exceeding 0.9 were considered well performing [52]. The OR performance threshold was set to below 0.15. All performance metrics were generated using the internal test data from cross-validation and then averaged across the five folds [51]. Binary models were made using a threshold optimised for TSS.

An independent tick occurrence dataset for validation was acquired through a pan-European tick surveillance projects supported by regional MSD Animal Health divisions [55, 56]. Veterinary practices across Europe were asked to submit ticks found on pets and record their geographic location (for details, see other papers in this volume). These surveillance projects ran for varying periods between 2015 and 2022 in 15 European countries: Austria, Belgium, Czech Republic, Denmark, Finland, France, Germany, Hungary, Italy, The Netherlands, Norway, Poland, Portugal, Romania, Slovakia, Spain and UK [57–59]. Ticks were then morphologically or molecularly identified by the study co-authors. This independent dataset was thinned so that no points were closer than 30 km and resulted in 570 occurrence points for *I. ricinus* and 133 for *D. reticulatus*. The same performance metrics used in the internal validation dataset were employed here.

An uncertainty index was generated by finding the range between the minimum and maximum value of predicted environmental suitability for each cell within the five replicates for each modelling combination. This was to show the uncertainty in the results derived from different subsets of occurrence data. The sum of the uncertainty was then normalised between 0 and 1 to allow for comparison.

Although quantitative analysis can provide a good estimation of the value of the model, expert qualitative analysis of the resultant maps is also a useful guide [60]. Here, the top performing model outputs, according to the performance metrics, were further critiqued by each of the co-authors and their national research groups to offer local knowledge.

#### Comparison of models

The performance metrics of different modelling approaches were compared statistically using a non-parametric Friedman test [61]. For each of the models built using different modelling algorithms, training extents and explanatory variables, the statistical difference in the AUC, TSS, OR, MCS and CBI, uncertainty index and the proportion of Europe considered suitable based on binary maps produced using the TSS optimised threshold were tested to determine whether the different input variables and modelling algorithms are equal in performance. Following this, a Dunn’s post hoc analysis, with Bonferroni correction of *P*-values, allowed for pairwise comparisons of models [62].

## Results

### Occurrence data

The combination of occurrence data from the literature reviews, GBIF data and additional publications provided 7668 georeferenced locations of *I. ricinus* and 2733 for *D. reticulatus* (Fig. 1). After cleaning, the datasets were reduced to 4908 and 858 records, respectively (Additional file 1). Finally, following the removal of spatial bias, the resultant datasets consisted of 638 locations for *I. ricinus* (optimal thinning; 50 km, NNI = 0.996, *Z* = − 0.176) and 153 for *D. reticulatus* (optimal thinning; 60 km, NNI = 1.014, *Z* = 0.337) (Additional file 1).

### Model performance

**Fig. 2:**
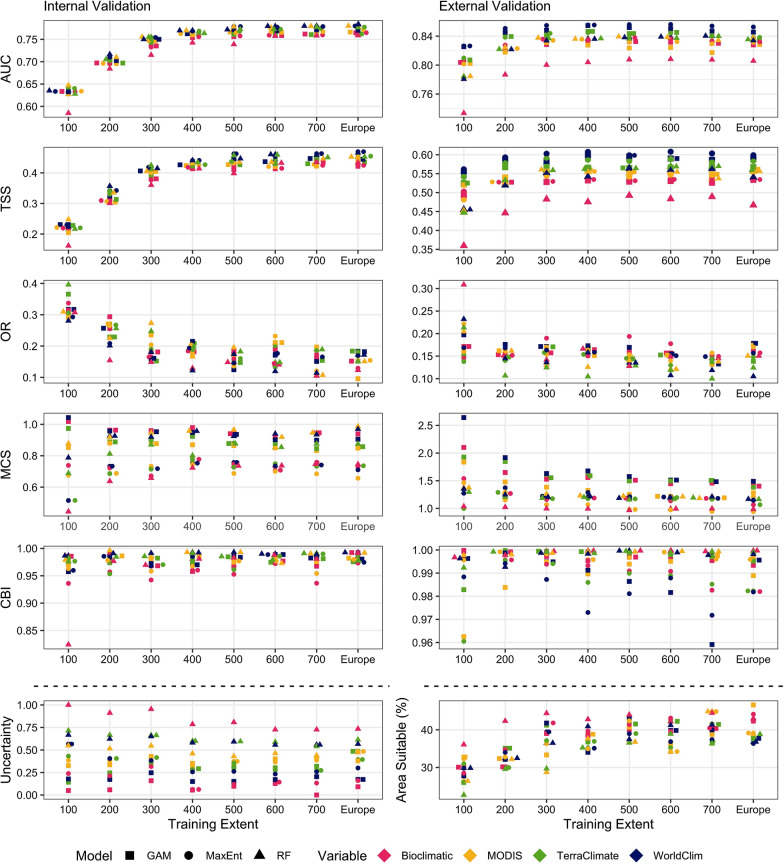
Comparison of model performance metrics for *Ixodes ricinus* using three modelling algorithms [generalised additive models (GAM), maximum entropy (MaxEnt) and random forests (RF)], with four explanatory variable sets (bioclimatic variables, WorldClim, TerraClimate and MODIS satellite-derived variables) and with eight training extents (100 km–700 km buffering extents around occurrence data increasing in increments of 100 km and the European extent). The area under the receiver-operating characteristic curve (AUC), true skill statistic (TSS), omission rate (OR), Miller’s calibration slope (MCS) and Continuous Boyce Index (CBI) were derived from the mean values from cross-validation folds. The uncertainty index represents the range in predictions between folds and the area suitable shows the percentage of Europe predicted as suitable for the species using binary maps made using a threshold optimised for TSS. Note that the *y*-axes are different in each panel and the data points are jittered around the *x*-axis to allow for better visualisation

**Fig. 3:**
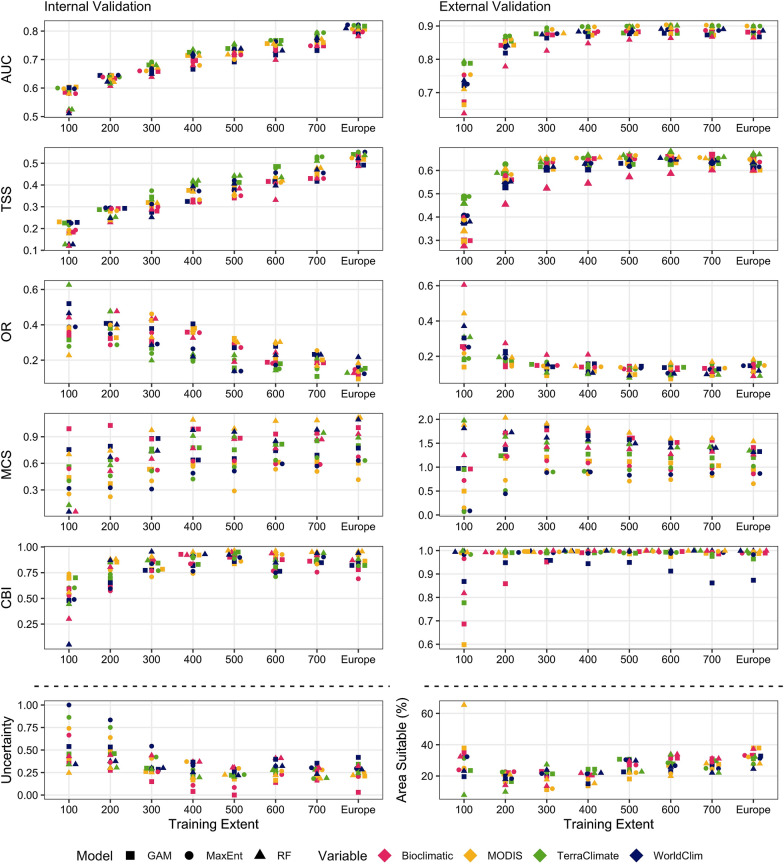
Comparison of model performance metrics for *Dermacentor reticulatus* using three modelling algorithms [generalised additive models (GAM), maximum entropy (MaxEnt) and random forests (RF)], with four explanatory variable sets (bioclimatic variables, WorldClim, TerraClimate and MODIS satellite-derived variables) and with eight training extents (100 km–700 km buffering extents around occurrence data increasing in increments of 100 km and the European extent). The area under the receiver-operating characteristic curve (AUC), true skill statistic (TSS), omission rate (OR), Miller’s calibration slope (MCS) and Continuous Boyce Index (CBI) were derived from the mean values from cross-validation folds. The uncertainty index represents the range in predictions between folds and the area suitable shows the percentage of Europe predicted as suitable for the species using binary maps made using a threshold optimised for TSS. Note that the *y*-axes are different in each panel and the data points are jittered around the *x*-axis to allow for better visualisation

**Fig. 4:**
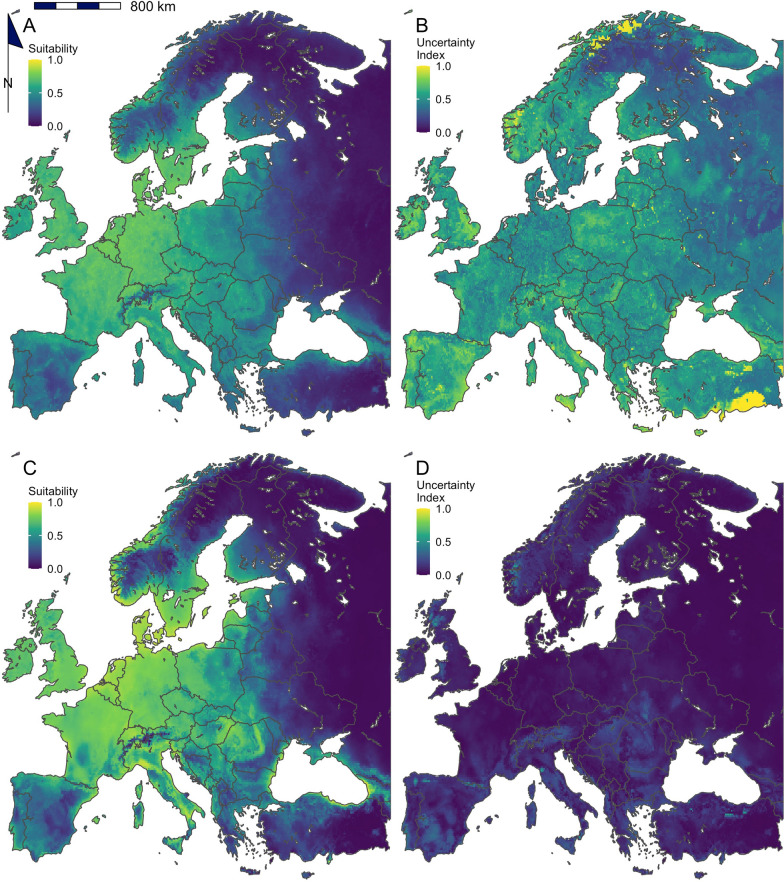
Mean predicted environmental suitability for *Ixodes ricinus* in Europe derived from 96 modelling approaches (**A**). The uncertainty in results between modelling approaches is also presented (**B**). The bottom graphics show the predicted environmental suitability for *I. ricinus* in Europe using a model trained with TerraClimate data from the extent of Europe and run using a MaxEnt modelling algorithm (**C**). The uncertainty in these results is also presented (**D**). This model was selected as best representing the current distribution of the species by a panel of experts; the output of all 96 models evaluated can be found in Additional file 7

**Fig. 5:**
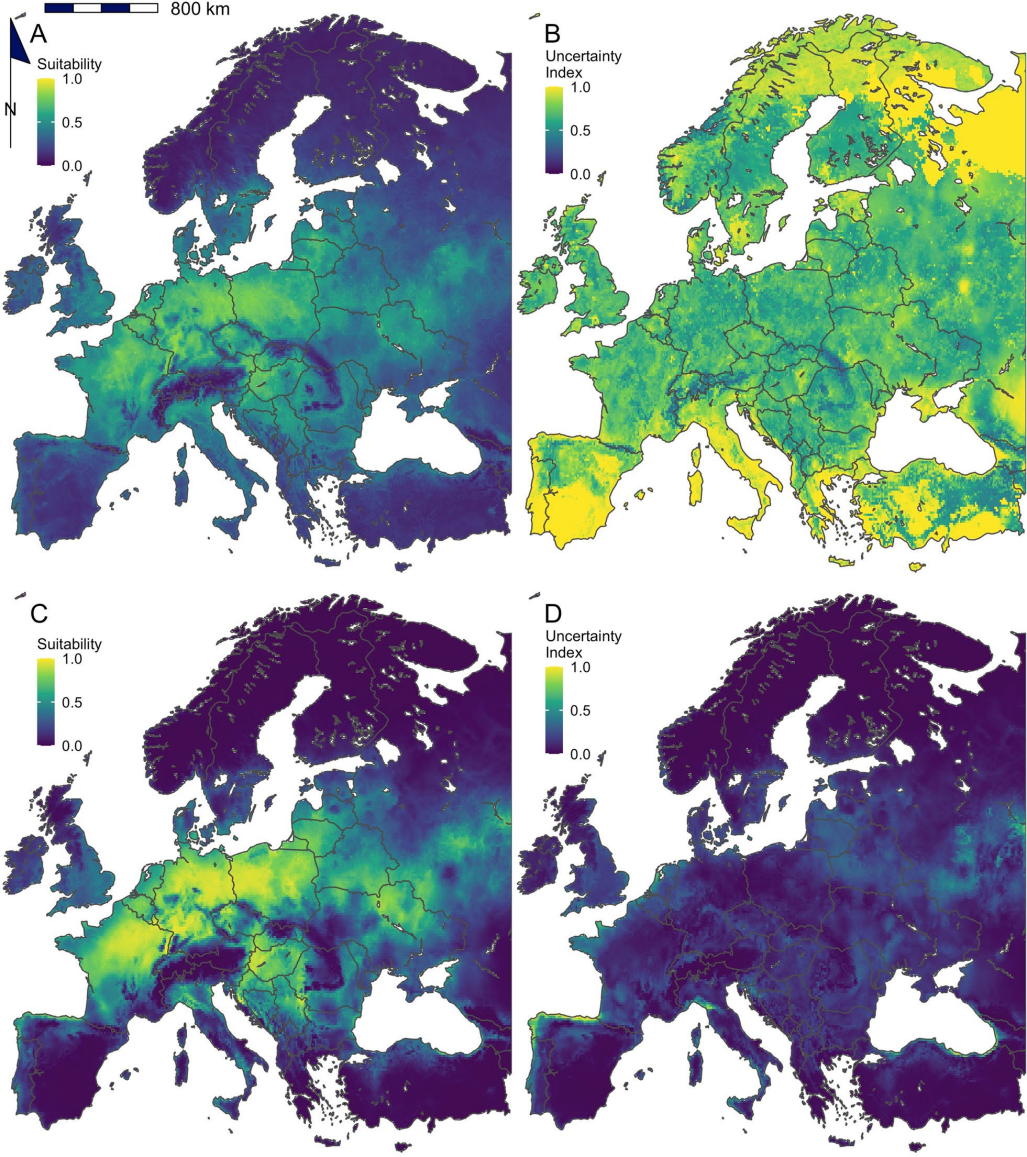
Mean predicted environmental suitability for *Dermacentor reticulatus* in Europe derived from 96 modelling approaches (**A**). The uncertainty in results between modelling approaches is also presented (**B**). The bottom graphics show the predicted environmental suitability for *D. reticultus* in Europe using a model trained with TerraClimate data from the extent of Europe and run using a MaxEnt modelling algorithm (**C**). The uncertainty in these results is also presented (**D**). This model was selected as best representing the current distribution of the species by a panel of experts; the output of all 96 models evaluated can be found in Additional file 8

Model performance varied widely, depending on the modelling algorithm, training extent and explanatory variables used, as well as the validation dataset. Furthermore, the different modelling combinations varied in their uncertainty index and the area of Europe predicted as suitable (Figs. 2, 3, 4 and 5) (Additional files 4, 5, 6, 7 and 8). The modelling approach also influenced the pattern of suitability across Europe. For *I. ricinus*, western Europe, specifically Germany, had the greatest consensus between modelling approaches and was always considered of high suitability, whereas western Russia had consistently low suitability values (Fig. 4). There were pockets of high variation in predicted *I. ricinus* suitability; for example, northern Scandinavia and southern Turkey had high uncertainty indexes. Central Ireland, southeast England and eastern regions in Spain were among those where suitability varied substantially depending on the modelling approach (Fig. 4) (Additional file 7). The results for *D. reticulatus* were similar, with Central Europe having high agreeability between different modelling approaches; Germany and Poland consistently had high suitability values. Similarly, the low suitability of regions of high altitude, such as the Alps, was consistent between methods (Fig. 5). However, there was greater and more widespread variation in predicted suitability for *D. reticulatus* compared to *I. ricinus*. Southern Europe varied significantly between methods, specifically southern Turkey, central Italy, northern Greece and southern Spain (Fig. 5) (Additional file 8).

#### Training extent

The training extent heavily influenced model performance for both *I. ricinus* (TSS: *X*^2^ = 419.90; *df* = 7; *P* < 0.005 | MCS: *X*^2^ = 72.40; *df* = 7; *P* < 0.005 | CBI: *X*^2^ = 32.12; *df* = 7; *P* < 0.005 | OR:* X*^2^ = 117.38; *df* = 7; *P* < 0.005) and *D. reticulatus* (TSS: *X*^2^ = 494.08; *df* = 7; *P* < 0.005 | MCS: *X*^2^ = 76.01; *df* = 7; *P* < 0.005 | CBI: *X*^2^ = 134.71; *df* = 7; *P* < 0.005 | OR: *X*^2^ = 185.14; *df* = 7 *P* < 0.005). Area under the ROC curve was not considered in the comparison of training extents as it is influenced by training extent [63]. When considering the internal validation, no models were adequate when using a 300-km buffer for *I. ricinus* or extents below a 500 km buffer for *D. reticulatus*. The training extent continued to influence the discrimination and classification performance of models positively for *D. reticulatus*, but performance plateaued at a 500-km buffering region for *I. ricinus*. However, with external validation, performance metrics peaked at around a 600-km buffering region for both species (Figs. 2, 3). The goodness of calibration metrics for *I*. *ricinus* and *D. reticulatus* improved with training extent, with the exception of *I. ricinus* internal validation. The variability in the CBI and MCS metrics between modelling approaches decreased with increasing training extent. The training extent also influenced the uncertainty index for *I. ricinus* (*X*^2^ = 33.75; *df* = 7; *P* < 0.005) and *D. reticulatus* (*X*^2^ = 58.28; *df* = 7; *P* < 0.005) model predictions. This effect was more obvious for *D. reticulatus* (Fig. 3). The proportion of Europe considered as suitable for each species changed with different training extents. For *I. ricinus*, there was a slight increase in suitable area with increasing extent (*X*^2^ = 53.94; *df* = 7; *P* < 0.005). For *D. reticulatus*, however, there was an initial decrease in predicted suitable area from a 100-km to 200-km buffering region, after which it increased (*X*^2^ = 50.08; *df* = 7; *P* < 0.005). The suitability maps produced using a 100-km training extent were not reflective of the distribution of *D. reticulatus*, with high suitability in northeast Scandinavia and Southern Spain (Additional file 8: Figure S13). Therefore, the analysis shows that the model training extent can impact model performance and predictions significantly.

#### Modelling algorithm

The effects of modelling algorithm on model performance were less pronounced than training extent (Figs. 2, 3). For *I. ricinus*, differences in the classification (TSS: *X*^2^ = 52.97; *df* = 2; *P* < 0.005) and discrimination (AUC: *X*^2^ = 61.54; *df* = 2; *P* < 0.005) results for internal validation were less obvious than external, but RF consistently performed worse than MaxEnt and GAM (Fig. 2). However, this difference was most obvious when assessing the uncertainty index (*X*^2^ = 49.56; *df* = 2; *P* < 0.005). Despite performing better, there were still differences in the resultant suitability maps between models generated using either GAM or MaxEnt, with the suitability of whole countries, such as Ireland, differing with each modelling algorithm (Additional file 5: Figure S10). The goodness of calibration of *I. ricinus* models also significantly varied depending on the modelling algorithm (MCS: *X*^2^ = 334.21; *df* = 2; *P* < 0.005 | CBI: *X*^2^ = 135.13; *df* = 2; *P* < 0.005) with RF performing significantly better than GAM. However, the poor classification, discrimination and uncertainty performance of RF was mirrored in the suitability maps produced, where output was oversimplified (Additional file 5: Figure S5-12). Compared to *I. ricinus*, differences in the classification (TSS: *X*^2^ = 29.96; *df* = 2; *P* < 0.005) and discrimination (AUC: *X*^2^ = 65.83; *df* = 2; *P* < 0.005) performance of the modelling algorithms were less evident for *D. reticulatus*, although RF models were still among some of the worst performing (Fig. 3). However, when assessing the MCS and CBI metric (MCS: *X*^2^ = 352.93; *df* = 2; *P* < 0.005 | CBI: *X*^2^ = 145.33; *df* = 2; *P* < 0.005), MaxEnt and GAM were the worst performing modelling algorithms, respectively. MaxEnt also had higher uncertainty indexes at smaller training extents (*X*^2^ = 12.06; *df* = 2; *P* < 0.005) and the resultant suitability maps were inaccurate with the northeastern regions predicted as highly suitable to *D. reticulatus* (Fig. 3) (Additional file 8: Fig S13). The modelling algorithm can have a pervasive influence on the performance of SDM.

#### Explanatory variables

The classification and discrimination performance of models was significantly influenced by the explanatory variables for both *I. ricinus* (AUC: *X*^2^ = 250.64; *df* = 3; *P* < 0.005 | TSS: *X*^2^ = 278.39; *df* = 3; *P* < 0.005) and *D. reticulatus* (AUC: *X*^2^ = 158.09; *df* = 3; *P* < 0.005 | TSS: *X*^2^ = 95.24; df = 3; P < 0.005). However, the goodness of calibration of *I. ricinus* (OR:* X*^2^ = 4.25; *df* = 3; *P* = 0.24 | CBI: *X*^2^ = 7.53; *df* = 3; *P* = 0.06 | MCS: *X*^2^ = 92.43; *df* = 3; *P* < 0.005) and *D. reticulatus* (OR: *X*^2^ = 11.39; *df* = 3; *P* = 0.01 | CBI: *X*^2^ = 18.49; *df* = 3; *P* < 0.005| MCS: *X*^2^ = 11.41; *df* = 3; *P* = 0.01) models was less influenced by explanatory variables. Overall, *I. ricinus* models built with interpolated WorldClim data performed best, statistically, in both internal and external validation, although qualitative interpretation of the resultant maps suggests that TerraClimate variables were able to predict the suitability best (see below: Expert Opinion). Models trained with TerraClimate variables performed best, statistically, for *D. reticulatus*. For both species, models built with bioclimatic variables were consistently among the lowest performing, especially when used in conjunction with RF (Figs. 2, 3). They also predicted the highest proportion of Europe as suitable for *I. ricinus*. For both *I. ricinus* (*X*^2^ = 9.05; *df* = 3; *P* = 0.03) and *D. reticulatus* (*X*^2^ = 11.35; *df* = 3; *P* = 0.01), the areas predicted as suitable were influenced by the explanatory variables, as each set captured different portions of their niches (Additional file 7, 8). For both species, maps produced with MODIS variables had a coarser appearance, while the suitability of ticks using the interpolated datasets had a smoother gradient of suitability across Europe. Models trained with MODIS variables also had an artefactual line across northern Scandinavia, due to cloud, ice and snow cover, which interrupted suitability predictions [64, 65].

#### Expert opinion

When assessing the resultant suitability maps of the best fit models, the consensus from expert opinions was that, although several modelling combinations performed well in general terms, none of the maps reflected the current distribution of either species in its entirety. This was most apparent for models generated with RF, which had oversimplified results that did not reflect the distribution of *I. ricinus,* particularly at the geographic margins of each species’ distribution, such as the south of Spain and northern Scandinavia (Additional file 7). These deviations were also evident in the models with *D. reticulatus* (Additional file 8).

The models which were considered by the experts to be most appropriate to describe *I. ricinus* and *D. reticulatus* are presented in Figs. 4 and 5 as well as the mean suitability derived from all 96 modelling approaches. Models trained with TerraClimate data from the extent of Europe and run using a MaxEnt modelling algorithm best described the current distribution of both *I. ricinus* and *D. reticulatus*. For *I. ricinus*, large regions of Central and Western Europe were considered suitable with decreasing suitability towards the Northern and Eastern Regions. There were limitations in the ability to capture the distribution for particular regions; for example, the suitability in Eastern Finland is likely greater than presented, as well as the northern region of Spain. For *D. reticulatus,* the area predicted as suitable is more conservative compared to their expected range, especially in Southern Europe. However, the high predicted climatic suitability in Germany and Poland accurately reflects the high abundance of *D. reticulatus* in these regions.

## Discussion

This study aimed to compare multiple modelling approaches in their ability to capture the niche of two important tick species in Europe, *I. ricinus* and *D. reticulatus,* to assess their potential usefulness in predicting spatial variability in the climate suitability. Using an array of modelling approaches and datasets, and three validation/verification methods, it was possible to identify the most useful model for each species, balancing statistical performance and plausibility. However, the overall evaluation showed that the performance and predictive ability of SDMs is highly dependent on the modelling algorithm and input variables and, although some models were broadly accurate in some regions, there was limited success in defining the species distribution to a localised level using any modelling approach (Figs. 2, 3, 4 and 5). This work also demonstrates the importance of using different validation techniques when assessing the overall performance of SDMs.

The predictive performance of each modelling approach was dependent on the validation technique used. There were differences when considering the internal vs. external validation here, showing the importance of considering both. Independent validation provides the benefit of assessing the predictive ability of the model against an external dataset, but this is often omitted from SDM studies [55, 66, 67]. The use of reliable citizen science may assist in the generation of more independent datasets for SDM validation [55]. Furthermore, as shown here (especially for RF models), models may perform well statistically, but the resultant accuracy of the maps generated may be poor. Inspection of the predictive performance of the model by local experts can be helpful in this respect [60]. The uncertainty index also highlighted regions of unreliability in the predictions. Model evaluation therefore should be based on multiple validation metrics and techniques, adding confidence to SDM conclusions.

The influence of modelling algorithm shows that there is not necessarily an ideal approach for all species. Within SDM literature, MaxEnt is often used without considering alternatives [68]. However, recent interest in this area has shown that the choice of algorithm can heavily influence the predictive ability of the SDM, and this is dependent on the species occurrence data and training extent [17, 42]. For example, here RF produced plausible predictions for *D. reticulatus* but overfitted and underestimated suitability of Europe to *I. ricinus* despite reasonable performance metrics. Interestingly, RF was the best performing algorithm when using SDM for estimating suitability for *D. variabilis* in the USA [42]. Researchers considering the use of SDM should compare several modelling algorithms for the species of interest and study region to ensure an appropriate one is used.

The spatial extent used in model training greatly influences model outputs [18, 37, 63]. The area the species has access to, in biogeographic history terms, is the most appropriate for SDM as theoretically all suitable environments should be occupied [69]. However, the delimitation of this area is often unknown and consequently different training extents have been used in tick SDM [70, 71]. The variation in predictions from models trained with different training extents in the present study reflects previous work whereby narrow or excessively broad training extents can decrease model performance [18, 37]. The Europe training extent, although not the largest used here, achieved good performance metrics and suitability maps, suggesting that European-wide modelling is realistic for *I. ricinus* and *D. reticulatus* species. Ticks are largely dispersed by their hosts; hence, it is likely they have successfully occupied most of the suitable regions in Europe [72]. This appears to be true for *I. ricinus*. However, rapid recent range expansion of *D. reticulatus* in central Europe and the patchy distribution of this species in some parts of its range suggest that this may not be the case for *D. reticulatus* [5, 10] (Fig. 1).

When using environmental variables, there is an assumption that the data are free from statistical error and fully capture the species’ niche [33]. However, both satellite-derived and interpolated data have limitations, as well as advantages. First, although there has been a recent increase in the use of satellite imagery in vector and vector-borne disease modelling, it often has a short temporal range (2000–present) and contains artefacts due to cloud, ice and snow cover [64, 65] (Additional files 7, 8). In contrast, the interpolated datasets are easily downloaded, span a greater temporal range (1970—present depending on the source) and are free from the aforementioned artefacts [73–75]. Furthermore, interpolated datasets have a greater range of variables available which can be used to describe the environmental constraints on the species, such as vapour pressure and deficit [8, 10]. The main limitation of interpolated data is that it is derived from networks of ground weather stations and hence there are inherent issues associated with collinearity [76]. Consequently, the results of models built with interpolated data should be viewed with caution, especially where no attempt has been made to reduce collinearity.

Despite multiple modelling algorithms, training extents and explanatory variables, none of the models produced reflected the current distribution of *I. ricinus* or *D. reticulatus* accurately across their entire known ranges. Many of the models were adequate according to performance metrics and the general pattern of suitability matched the recognised distribution of each species, but there were localised discrepancies between predicted suitability and known distributions. These were also species specific, with the resultant maps being less representative for *D. reticulatus*, possibly because of its narrower spatial range and fragmented distribution throughout Europe, making it harder to capture its complex niche [5, 10]. This, and the possibility that species may adapt to local climatic conditions (e.g. as has been demonstrated with *I. ricinus* [77, 78]), suggests that if researchers require more localised, sensitive tick predictions, regional SDM may be more appropriate [14, 79, 80]. Nevertheless, the validation completed here demonstrates that broadly accurate models can be developed for larger regions, which may be useful for larger scale climate impact assessments.

The use of singular climatic datasets, such as those used here, may not fully capture the environmental niche of the ticks. Although relative humidity and temperature are key variables influencing tick presence, habitat and host variables are also important determinants [79, 80]. For example, the sandy west coast of Denmark had greater predicted suitability for *I. ricinus* compared to the wooded eastern regions of Denmark when models were trained with bioclimatic variables, which is the opposite of the known distribution of *I. ricinus* in this region [79]. These local discrepancies could result from the lack of habitat variables. The inclusion of additional variables such as host distribution or abundance, vegetation cover and soil type may increase the accuracy of SDM at local levels [79, 80]. However, these data are often not freely available at high resolution across the whole extent of Europe and, more importantly, cannot be used in predicting future suitability because of the uncertainty in anthropogenic changes, extreme weather events and host distributions [81–83].

As SDMs work by defining the species occurrence dataset in environmental spaces, inaccuracies in this dataset may cause erroneous results. The misidentification of ticks and the emergence of new species, which closely resemble well-established species, such *I. inopinatus*, introduce ambiguity into the historical records [84, 85]. A recent study showed that researchers in the Western Palearctic and North Africa misidentified 29.6% of ticks [86]. An alternative source of error is through the documentation of ticks from migrating hosts which do not represent permanent populations. For example, *Hyalomma* species have been introduced to the UK on migratory birds and including such data would distort the species niche in environmental space [87]. Furthermore, these models rely on pseudo-absence data as collecting true absence data for ticks is likely to cause type II statistical errors (false negatives) [31]. Collecting ticks depends on sampling method, time of collection and weather conditions; hence, their absence is not always representative of an unsuitable habitat [88]. The inability of these climate models to effectively capture the species niche may in part be due to subtle errors in occurrence data and the lack of true absence data for ticks.

## Conclusion

SDMs are powerful tools in identifying the suitability of environmental spaces to species of interest, such as ticks. However, despite considering multiple modelling approaches, there is no single modelling approach, using climatic variables alone, which can accurately capture the entire niche of *I. ricinus* or *D. reticulatus* throughout Europe. This is an important consideration when investigating the impacts of climate-mediated changes on tick distribution and the risk of tick-borne disease, since other factors such as host distribution, vegetation type, land use or other anthropogenic disturbance, are all likely to play critical roles within the broad habitat/climate envelope and should be included in a next-generation modelling approach.

### Supplementary Information

**Additional file 1: Dataset S1.** Species occurrence data for *Ixodes ricinus *and* Dermacentor reticulatus *used for model training (prior to thinning)*.*

**Additional file 2: Figure S1.** Principal component analysis decomposition of the contribution of each variable in the four datasets, bioclimatic variables (A), MODIS satellite-derived variables (B), WorldClim variables (C) and TerraClimate variables (D), which were used in determining the niche of *Ixodes ricinus*. The direction, length and colour of the arrows represent the contribution of each variable that was used in determining the niche of *Ixodes ricinus*.

**Additional file 3: Figure S2.** Principal component analysis decomposition of the contribution of each variable in the four datasets, bioclimatic variables (A), MODIS satellite-derived variables (B), WorldClim variables (C) and TerraClimate variables (D), which were used in determining the niche of *Dermacentor reticulatus*. The direction, length and colour of the arrows represent the contribution of each variable that was used in determining the niche of *Dermacentor reticulatus*.

**Additional file 4: Dataset S2.** The model performance metrics for 96 modelling approaches for *Ixodes ricinus* and *Dermacentor reticulatus*. Performance metrics include the area under the receiver-operating characteristic curve, true skill statistics, Miller’s calibration slope, continuous Boyce index and omission rate. The different modelling approaches included three modelling algorithms [random forests (RF), maximum entropy (MaxEnt) and generalised additive models (GAM)], with four explanatory variable sets (bioclimatic variables, WorldClim, TerraClimate and MODIS satellite-derived variables) and with eight training extents (100 km–700 km buffering extents around occurrence data increasing in increments of 100 km and the European extent).

**Additional file 5: Figure S3.** The statistical comparison of *Ixodes ricinus* model performance metrics using a Friedman test and post hoc Dunn’s test, with Bonferroni correction. The performance of the three modelling algorithms [random forests (RF), maximum entropy (MaxEnt) and generalised additive models (GAM)] (A–E), eight training extents (100 km–700 km buffering extents around occurrence data increasing in increments of 100 km and the European extent) (F–J) and four sets of explanatory variables (bioclimatic variables, WorldClim, TerraClimate and MODIS satellite-derived variables) (K–O) were compared using the area under the receiver-operating characteristic curve, true skill statistic, omission rate, Miller’s calibration slope, Boyce index, uncertainty index and the percentage of Europe considered as suitable for *Ixodes ricinus* in Europe. The text inside each cell shows the *P*-value of the Dunn’s test, and darker colours indicate lower *P*-values.

**Additional file 6: Figure S4.** The statistical comparison of *Dermacentor reticulatus* model performance metrics using a Friedman test and post hoc Dunn’s test with Bonferroni correction. The performance of the three modelling algorithms [random forests (RF), maximum entropy (MaxEnt) and generalised additive models (GAM)] (A–E), eight training extents (100 km–700 km buffering extents around occurrence data increasing in increments of 100 km and the European extent) (F–J) and four sets of explanatory variables (bioclimatic variables, WorldClim, TerraClimate and MODIS satellite-derived variables) (K–O) were compared using the area under the receiver-operating characteristic curve, true skill statistic, omission rate, Miller’s calibration slope, Boyce index, uncertainty index and the percentage of Europe considered as suitable for *Dermacentor reticulatus* in Europe. The text inside each cell shows the *P*-value of the Dunn’s test, and darker colours indicate lower *P*-values.

**Additional file 7: Figure S5-12.** The predicted environmental suitability for *Ixodes ricinus* in Europe using 96 different modelling approaches, using three modelling algorithms [random forests (RF), maximum entropy (MaxEnt) and generalised additive models (GAM)] with four explanatory variable sets (bioclimatic variables, WorldClim, TerraClimate and MODIS satellite-derived variables), with eight training extents (100 km–700 km buffering extents around occurrence data increasing in increments of 100 km and the European extent).

**Additional file 8: Figure S13-20.** The predicted environmental suitability for *Dermacentor reticulatus* in Europe using 96 different modelling approaches, using three modelling algorithms [random forests (RF), maximum entropy (MaxEnt) and generalised additive models (GAM)] with four explanatory variable sets (bioclimatic variables, WorldClim, TerraClimate and MODIS satellite-derived variables) with eight training extents (100 km–700 km buffering extents around occurrence data increasing in increments of 100 km and the European extent).

**Additional file 9: Text S1.** The ODMAP (overview, data, model, assessment, prediction) protocol for reporting species distribution modelling 90.

## Data Availability

Occurrence datasets used for model development are available in the supplementary material. The occurrence datasets and explanatory variable datasets used for model development and internal validation are available under a GPL-3 licence from [89]. Request to access and reuse the secondary datasets used for external validation must be made directly to the authors of the primary research. The ODMAP (Overview, Data, Model, Assessment, Prediction) protocol for reporting SDM was completed (Additional file 9: Text S1) [90]. The R scripts are available upon request to the corresponding or senior author.

## Abbreviations

GAM: Generalised additive models
MaxEnt: Maximum entropy
RF: Random forests
AUC: Area under the receiver-operating characteristic curve
TSS: True skill statistic
OR: Omission rate
SDM: Species distribution models
MCS: Miller’s calibration slope
CBI: Continuous Boyce index
