# Supplementary figures and images for: Predicting the distribution of Ixodes ricinus and Dermacentor reticulatus in Europe: a comparison of climate niche modelling approaches
Source: Parasit Vectors. 2023 Oct 25;16:384. doi: 10.1186/s13071-023-05959-y (PMC10601327; doi:10.1186/s13071-023-05959-y)

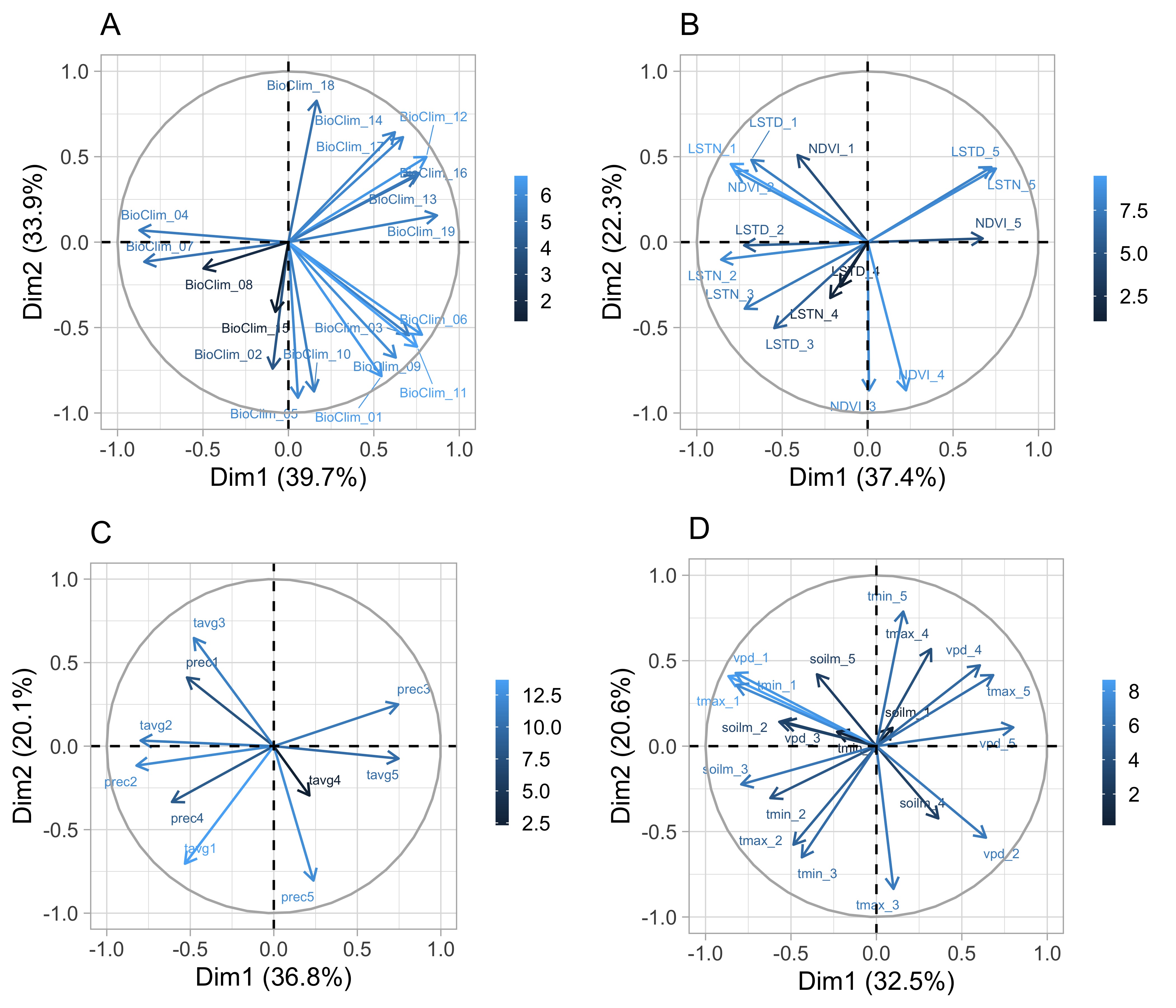

Supplement: Supplementary file 2 — Additional file 2: Figure S1. Principal component analysis decomposition of the contribution of each variable in the four datasets, bioclimatic variables (A), MODIS satellite-derived variables (B), WorldClim variables (C) and TerraClimate variables (D), which were used in determining the niche of Ixodes ricinus. The direction, length and colour of the arrows represent the contribution of each variable that was used in determining the niche of Ixodes ricinus. [file 13071_2023_5959_MOESM2_ESM.jpg]

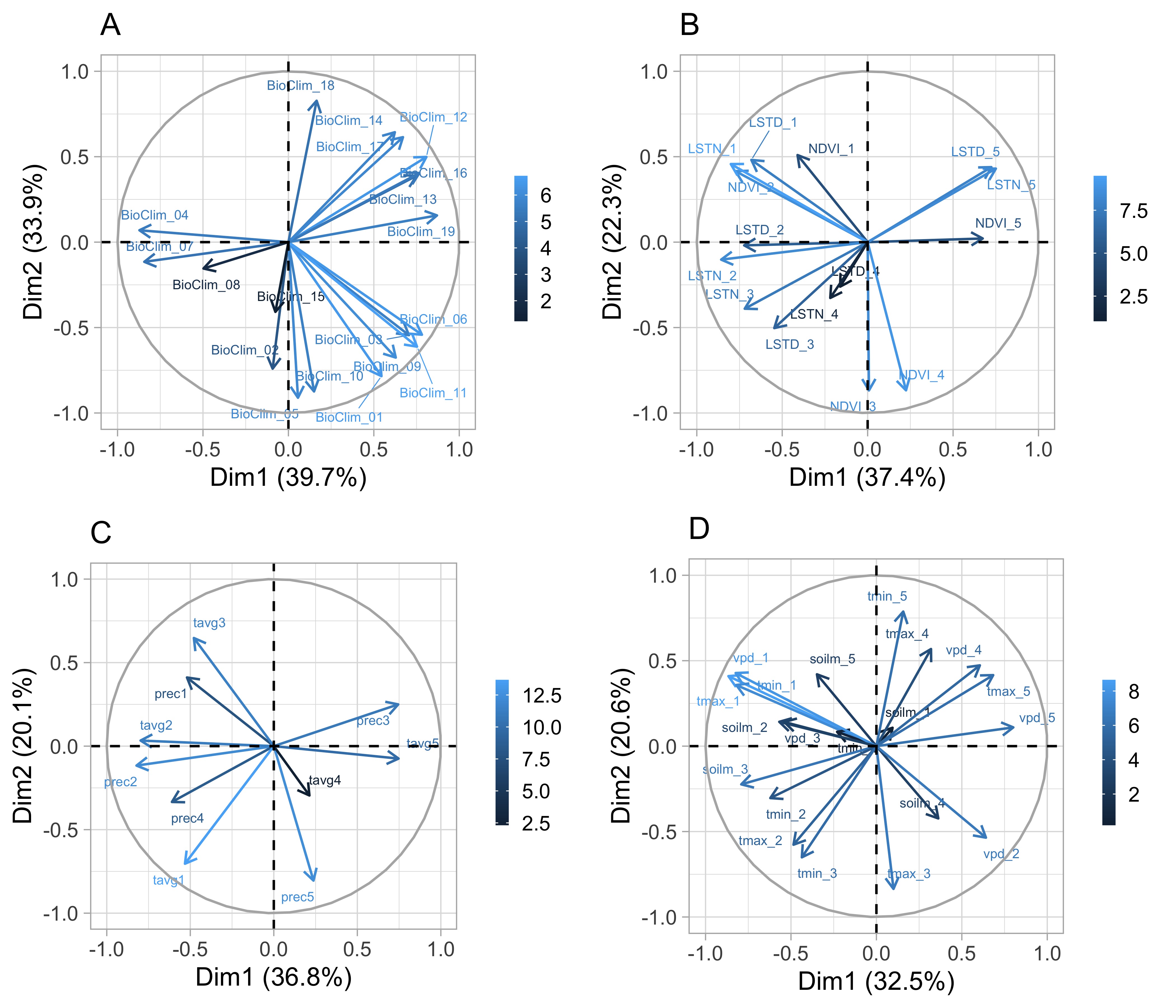

Supplement: Supplementary file 3 — Additional file 3: Figure S2. Principal component analysis decomposition of the contribution of each variable in the four datasets, bioclimatic variables (A), MODIS satellite-derived variables (B), WorldClim variables (C) and TerraClimate variables (D), which were used in determining the niche of Dermacentor reticulatus. The direction, length and colour of the arrows represent the contribution of each variable that was used in determining the niche of Dermacentor reticulatus. [file 13071_2023_5959_MOESM3_ESM.jpg]
