# Supplementary material for: Predicting the distribution of Ixodes ricinus and Dermacentor reticulatus in Europe: a comparison of climate niche modelling approaches
Source: Parasit Vectors. 2023 Oct 25;16:384. doi: 10.1186/s13071-023-05959-y (PMC10601327; doi:10.1186/s13071-023-05959-y)
Supplement: Supplementary file 7 — Additional file 7: Figure S5-12. The predicted environmental suitability for Ixodes ricinus in Europe using 96 different modelling approaches, using three modelling algorithms [random forests (RF), maximum entropy (MaxEnt) and generalised additive models (GAM)] with four explanatory variable sets (bioclimatic variables, WorldClim, TerraClimate and MODIS satellite-derived variables), with eight training extents (100 km–700 km buffering extents around occurrence data increasing in increments of 100 km and the European extent). [file 13071_2023_5959_MOESM7_ESM.docx]

**Additional File 7: Figure S5-12.** The predicted environmental suitability for *Ixodes ricinus* in Europe using 96 different modelling approaches, using three modelling algorithms [random forests (RF), maximum entropy (MaxEnt) and generalised additive models (GAM)], four explanatory variable sets (bioclimatic variables, WorldClim, TerraClimate and MODIS satellite-derived variables) and eight training extents (100 km – 700 km buffering extents around occurrence data increasing in increments of 100 km, and the European extent).


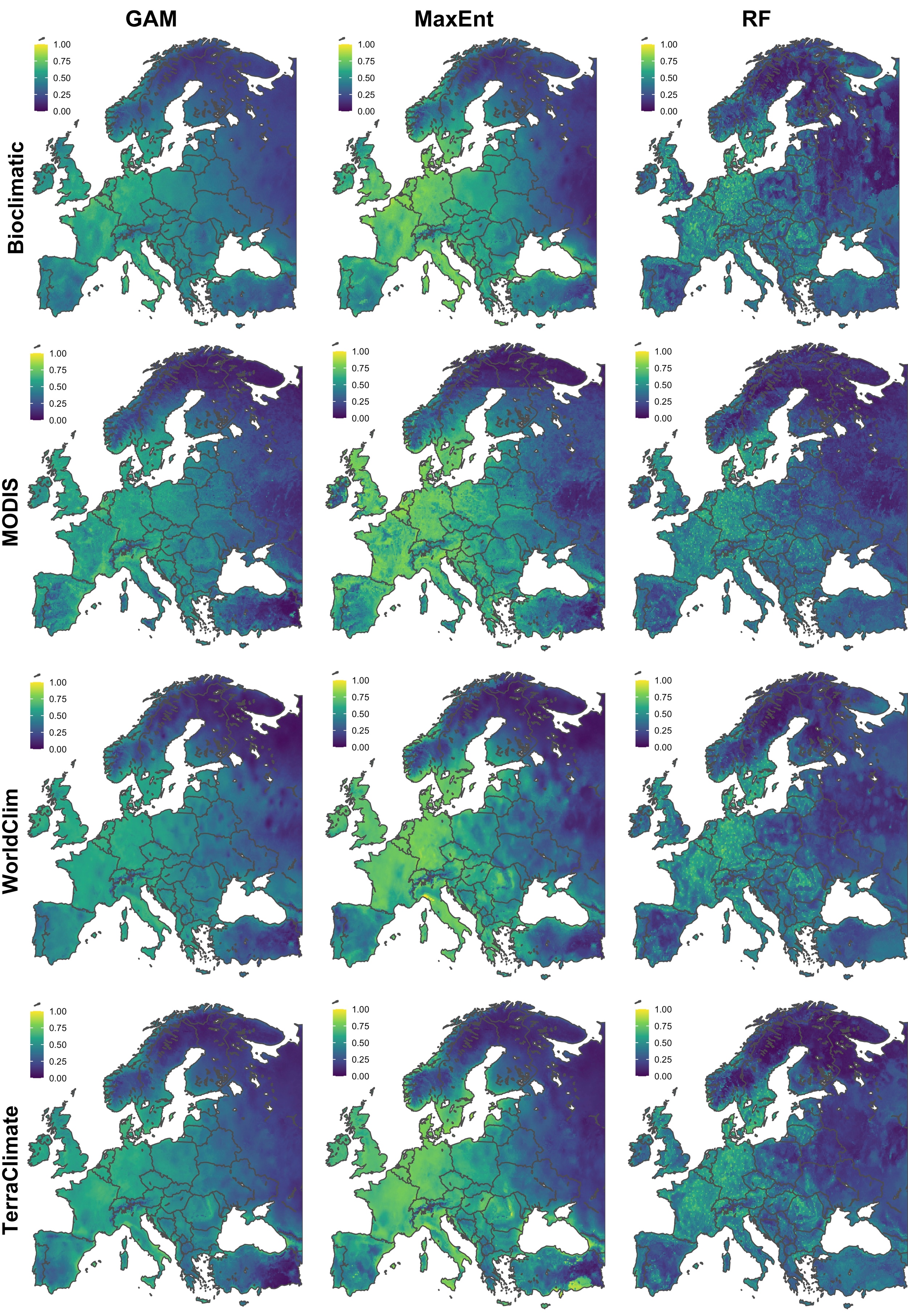


**Figure S5.** The predicted environmental suitability for *Ixodes ricinus* in Europe using different modelling approaches, including three modelling algorithms [random forests (RF), maximum entropy (MaxEnt) and generalised additive models (GAM)] and four explanatory variable sets (bioclimatic variables, WorldClim, TerraClimate and MODIS satellite-derived variables) using a 100km buffer training extent.


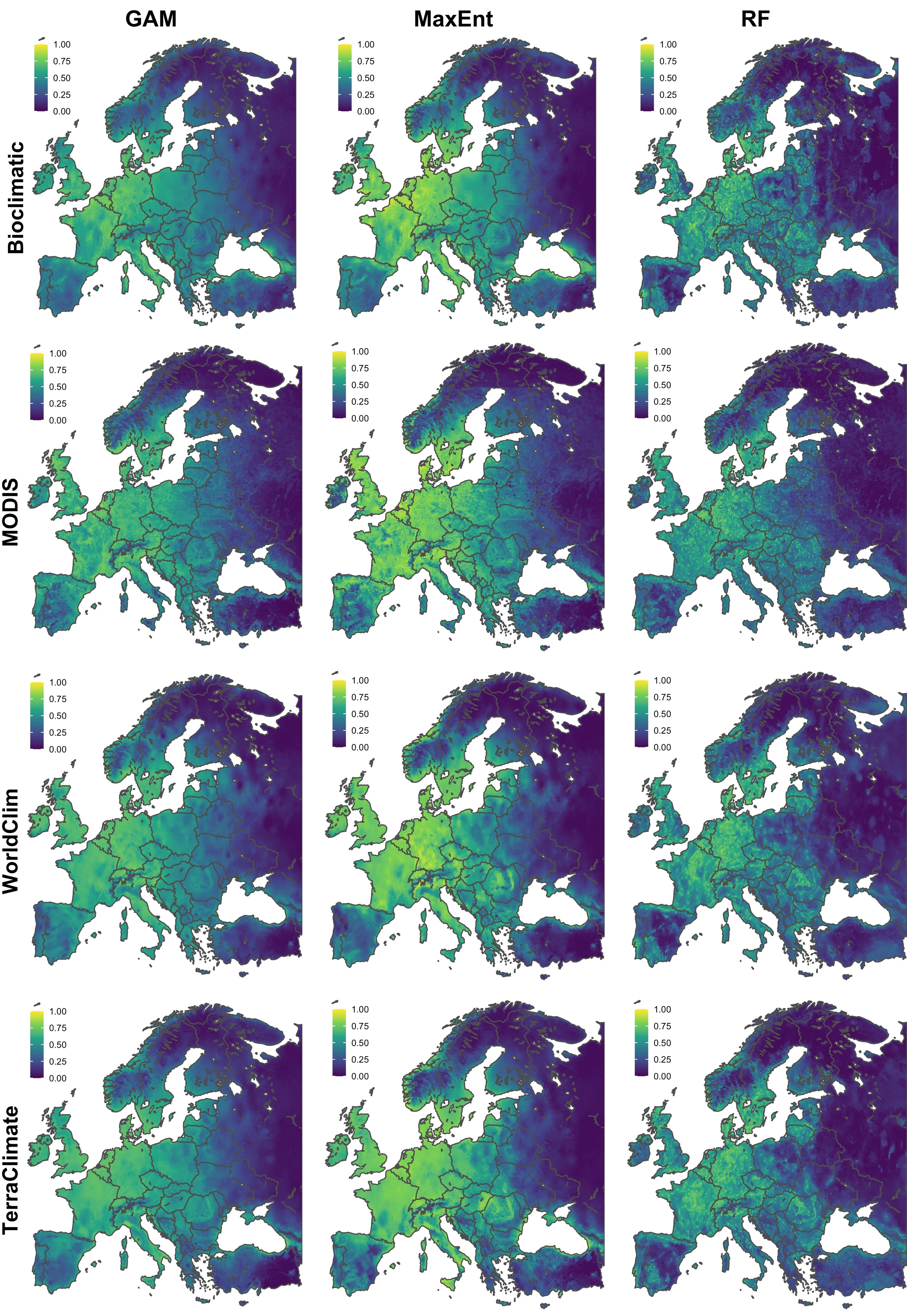


**Figure S6.** The predicted environmental suitability for *Ixodes ricinus* in Europe using different modelling approaches, including three modelling algorithms [random forests (RF), maximum entropy (MaxEnt) and generalised additive models (GAM)] and four explanatory variable sets (bioclimatic variables, WorldClim, TerraClimate and MODIS satellite-derived variables) using a 200km buffer training extent.


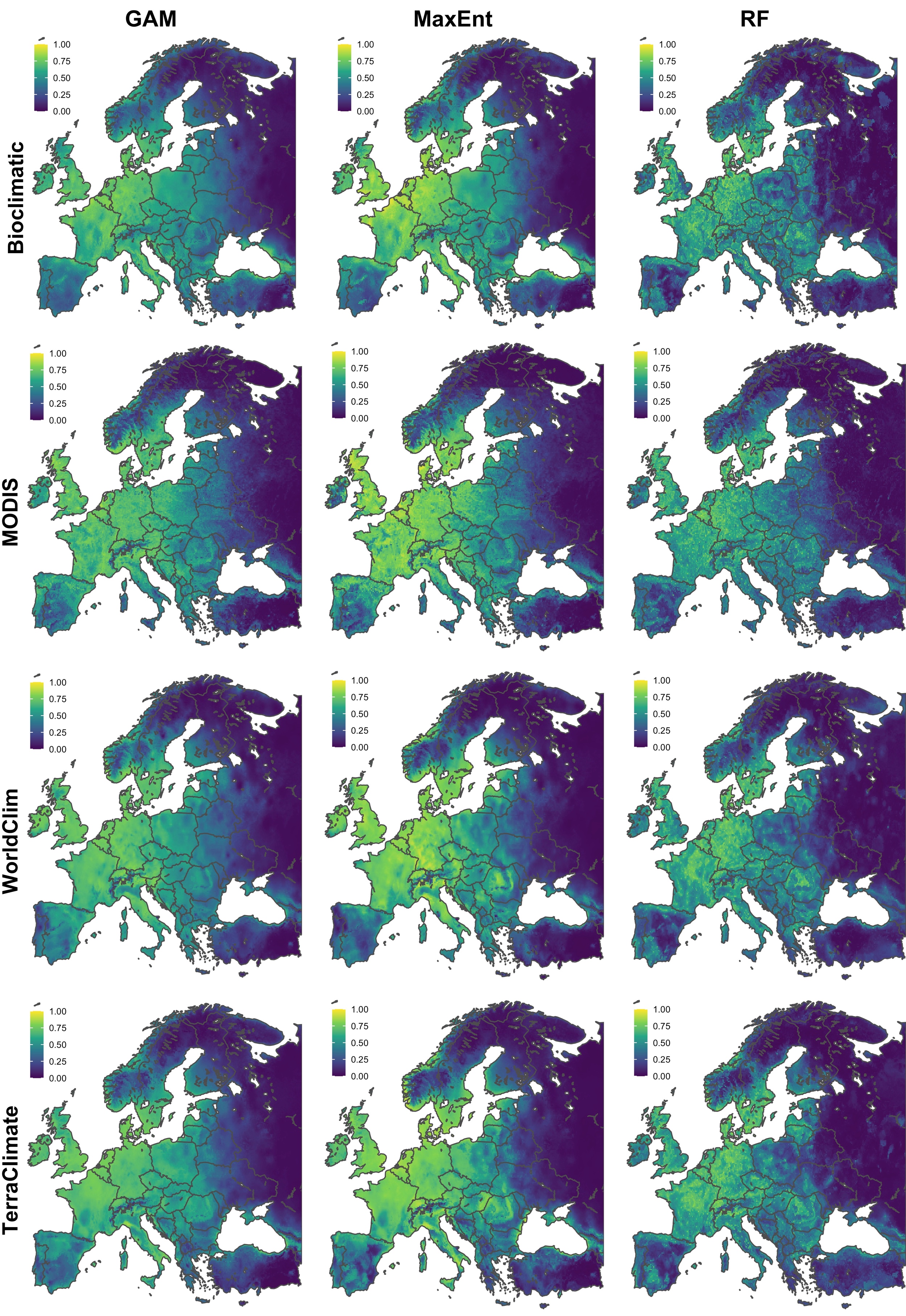


**Figure S7.** The predicted environmental suitability for *Ixodes ricinus* in Europe using different modelling approaches, including three modelling algorithms [random forests (RF), maximum entropy (MaxEnt) and generalised additive models (GAM)] and four explanatory variable sets (bioclimatic variables, WorldClim, TerraClimate and MODIS satellite-derived variables) using a 300km buffer training extent.


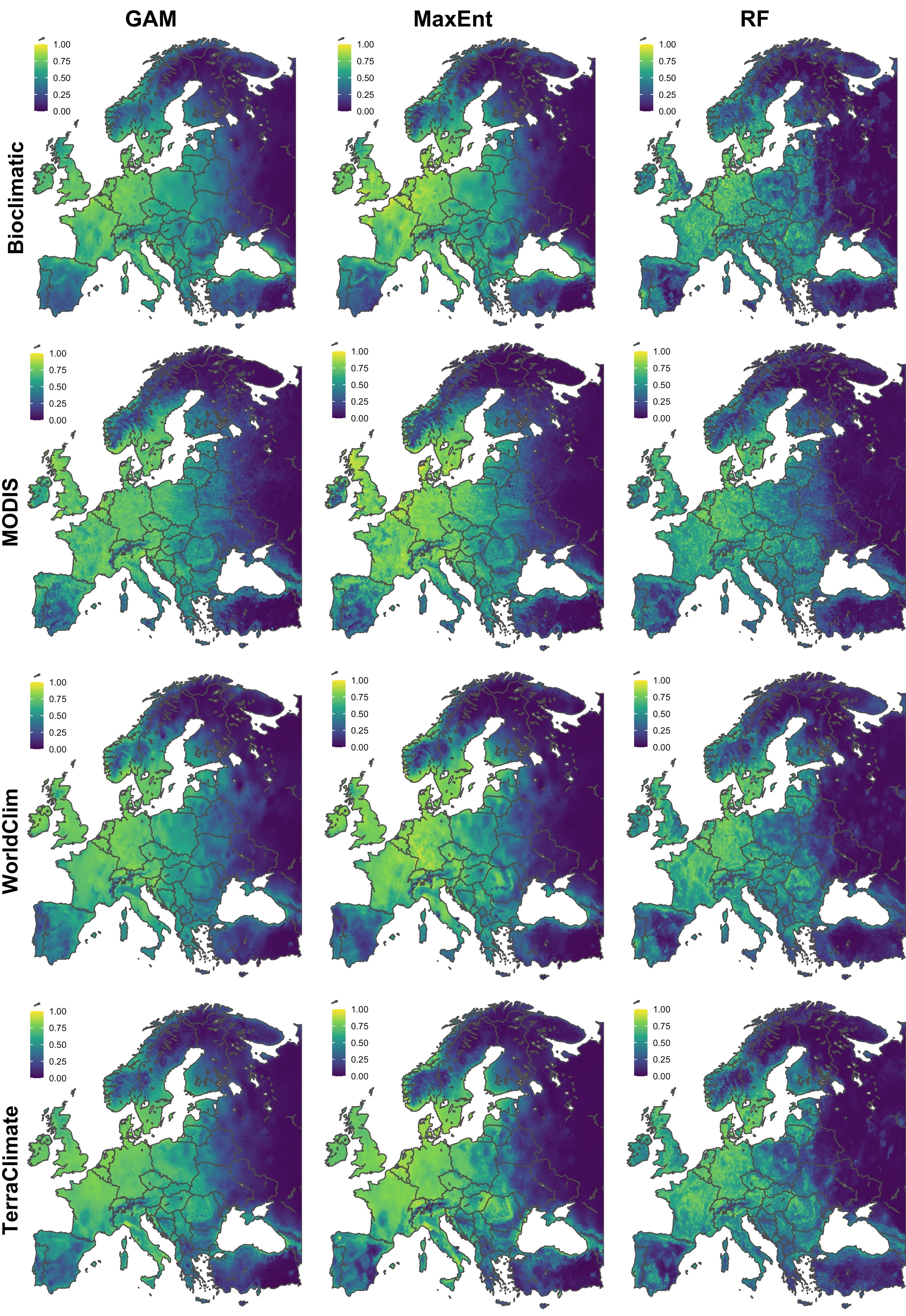


**Figure S8.** The predicted environmental suitability for *Ixodes ricinus* in Europe using different modelling approaches, including three modelling algorithms [random forests (RF), maximum entropy (MaxEnt) and generalised additive models (GAM)] and four explanatory variable sets (bioclimatic variables, WorldClim, TerraClimate and MODIS satellite-derived variables) using a 400km buffer training extent.


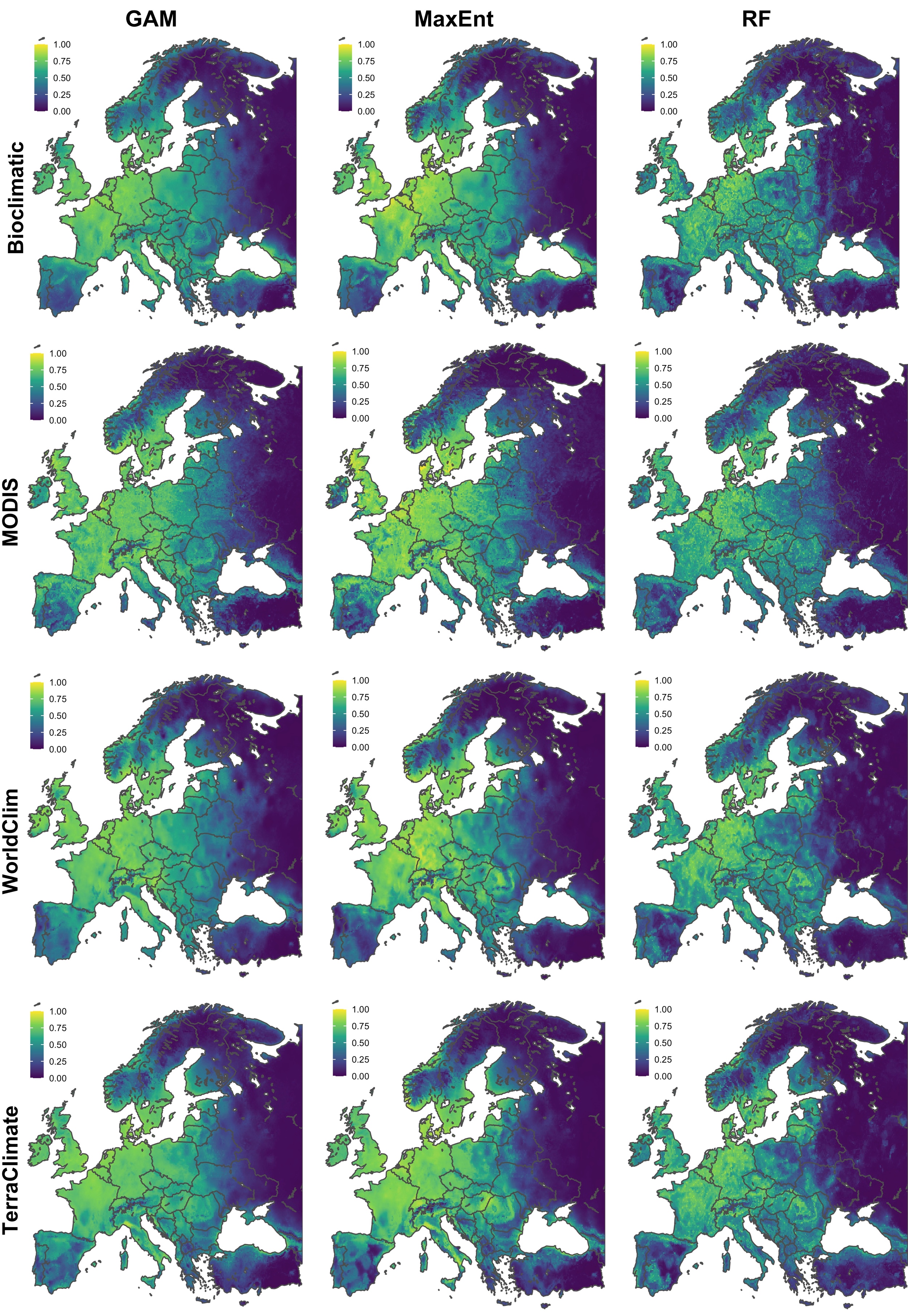


**Figure S9.** The predicted environmental suitability for *Ixodes ricinus in* Europe using different modelling approaches, including three modelling algorithms [random forests (RF), maximum entropy (MaxEnt) and generalised additive models (GAM)] and four explanatory variable sets (bioclimatic variables, WorldClim, TerraClimate and MODIS satellite-derived variables) using a 500km buffer training extent.


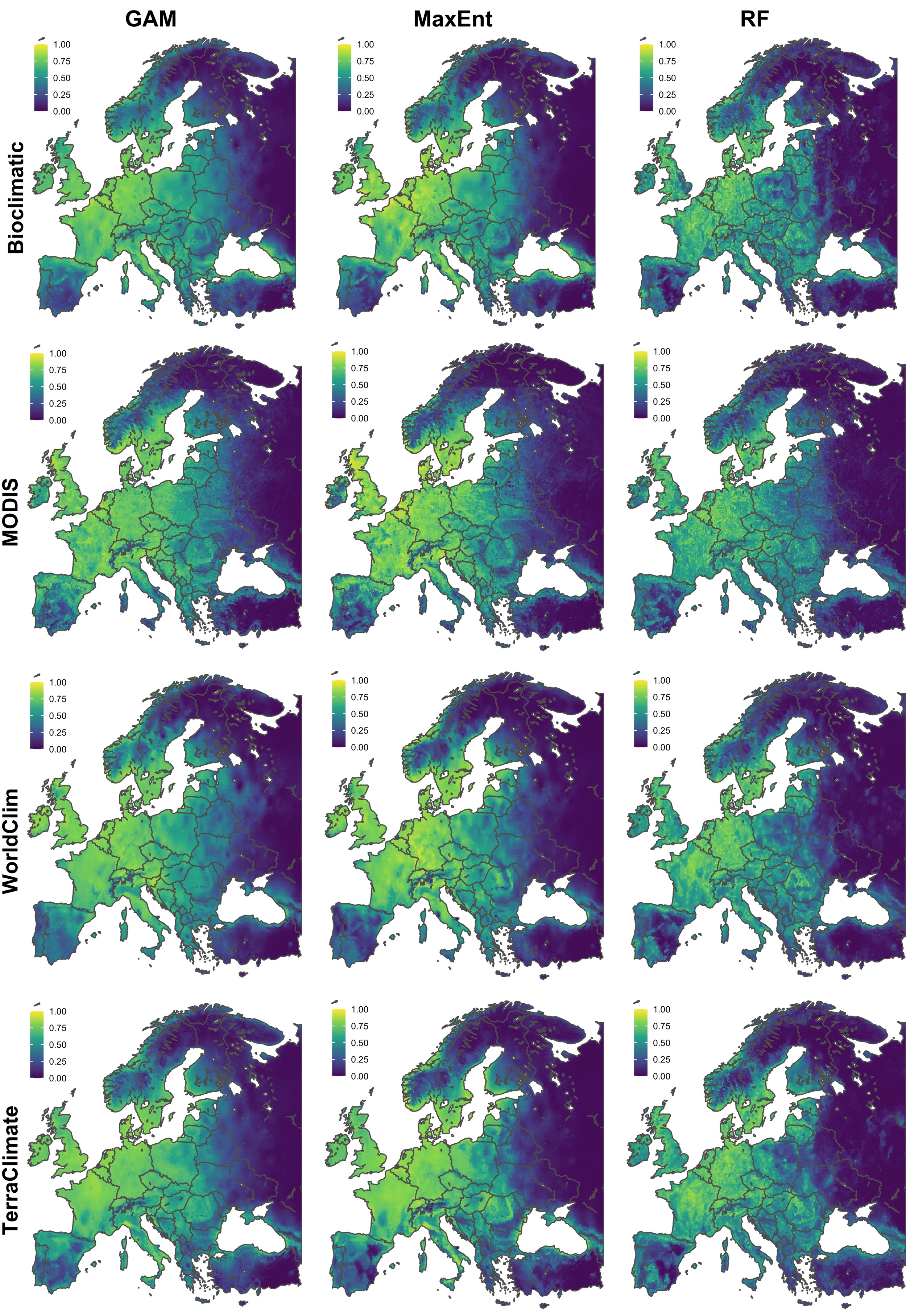


**Figure S10.** The predicted environmental suitability for *Ixodes ricinus in* Europe using different modelling approaches, including three modelling algorithms [random forests (RF), maximum entropy (MaxEnt) and generalised additive models (GAM)] and four explanatory variable sets (bioclimatic variables, WorldClim, TerraClimate and MODIS satellite-derived variables) using a 600km buffer training extent.


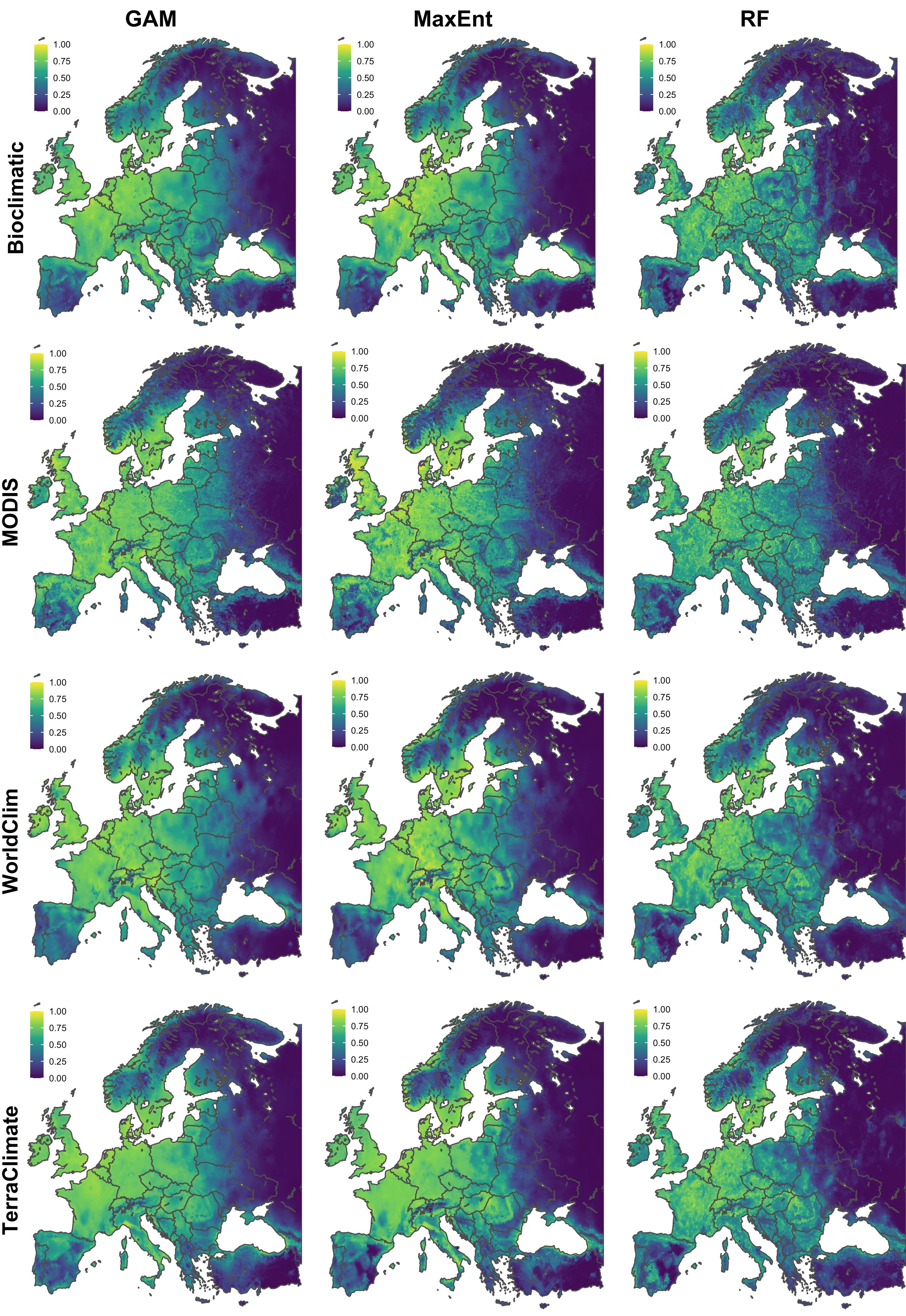


**Figure S11.** The predicted environmental suitability for *Ixodes ricinus* in Europe using different modelling approaches, including three modelling algorithms [random forests (RF), maximum entropy (MaxEnt) and generalised additive models (GAM)] and four explanatory variable sets (bioclimatic variables, WorldClim, TerraClimate and MODIS satellite-derived variables) using a 700km buffer training extent.


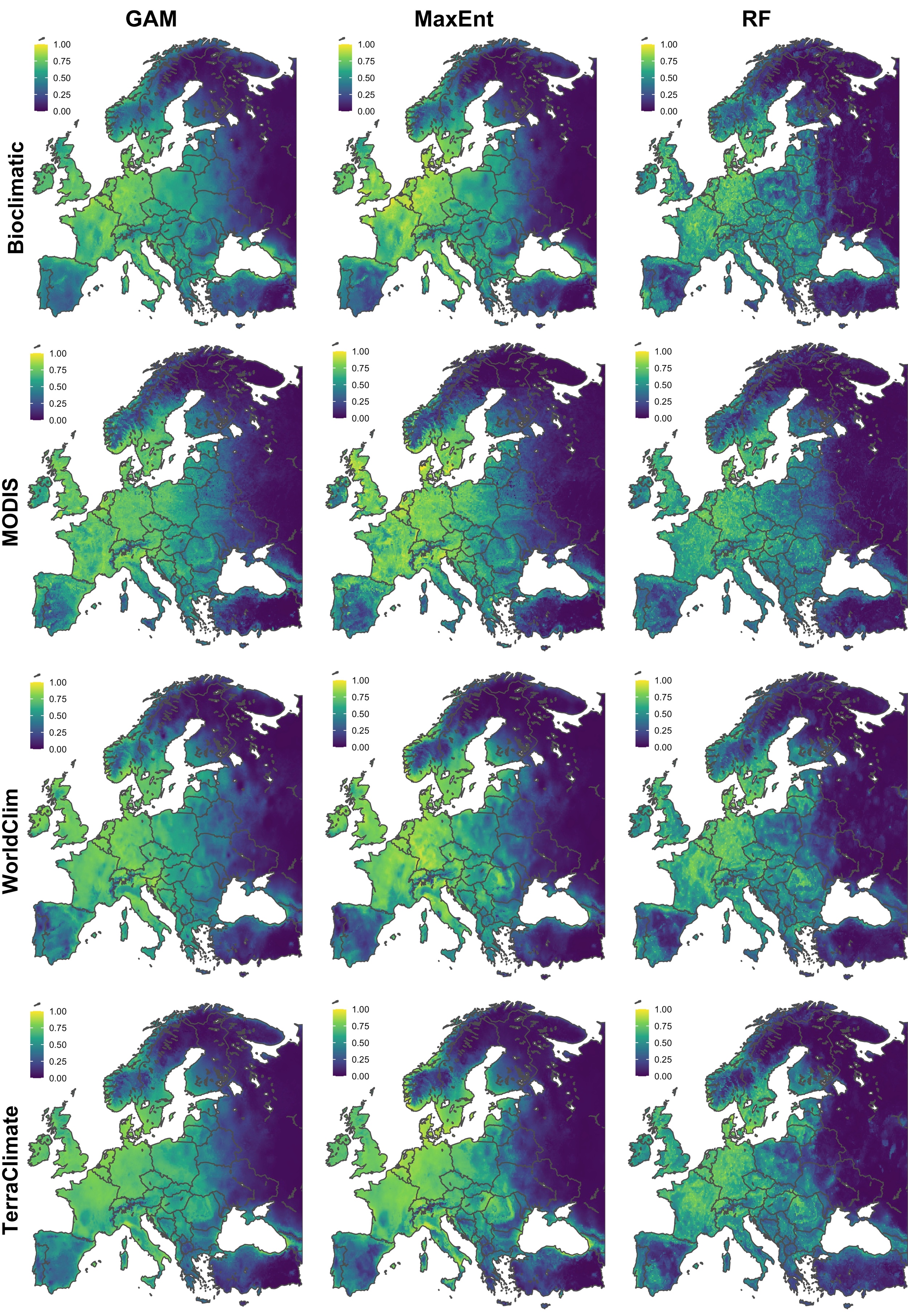


**Figure S12.** The predicted environmental suitability for *Ixodes ricinus* in Europe using different modelling approaches, including three modelling algorithms [random forests (RF), maximum entropy (MaxEnt) and generalised additive models (GAM)] and four explanatory variable sets (bioclimatic variables, WorldClim, TerraClimate and MODIS satellite-derived variables) using a training extent the size of Europe.
