# Supplementary material for: Predicting the distribution of Ixodes ricinus and Dermacentor reticulatus in Europe: a comparison of climate niche modelling approaches
Source: Parasit Vectors. 2023 Oct 25;16:384. doi: 10.1186/s13071-023-05959-y (PMC10601327; doi:10.1186/s13071-023-05959-y)
Supplement: Supplementary file 8 — Additional file 8: Figure S13-20. The predicted environmental suitability for Dermacentor reticulatus in Europe using 96 different modelling approaches, using three modelling algorithms [random forests (RF), maximum entropy (MaxEnt) and generalised additive models (GAM)] with four explanatory variable sets (bioclimatic variables, WorldClim, TerraClimate and MODIS satellite-derived variables) with eight training extents (100 km–700 km buffering extents around occurrence data increasing in increments of 100 km and the European extent). [file 13071_2023_5959_MOESM8_ESM.docx]

**Additional File 8: Figure S13-20.** The predicted environmental suitability for *Dermacentor reticulatus* in Europe using 96 different modelling approaches, using three modelling algorithms [random forests (RF), maximum entropy (MaxEnt) and generalised additive models (GAM)], four explanatory variable sets (bioclimatic variables, WorldClim, TerraClimate and MODIS satellite-derived variables)with eight training extents (100 km – 700 km buffering extents around occurrence data increasing in increments of 100 km, and the European extent).


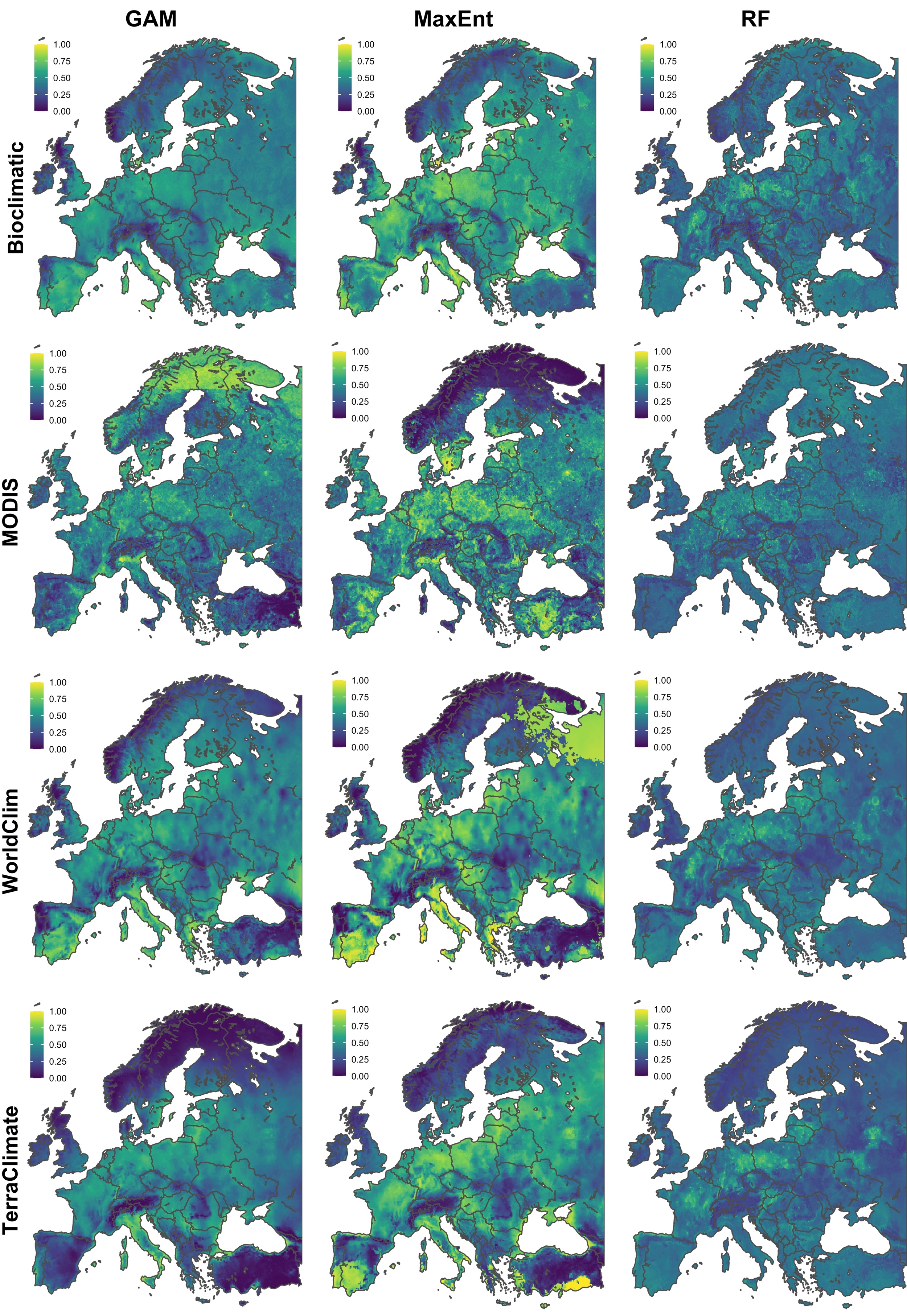


**Figure S13.** The predicted environmental suitability for *Dermacentor reticulatus* in Europe using different modelling approaches, including three modelling algorithms [random forests (RF), maximum entropy (MaxEnt) and generalised additive models (GAM)] and four explanatory variable sets (bioclimatic variables, WorldClim, TerraClimate and MODIS satellite-derived variables) using a 100km buffer training extent.


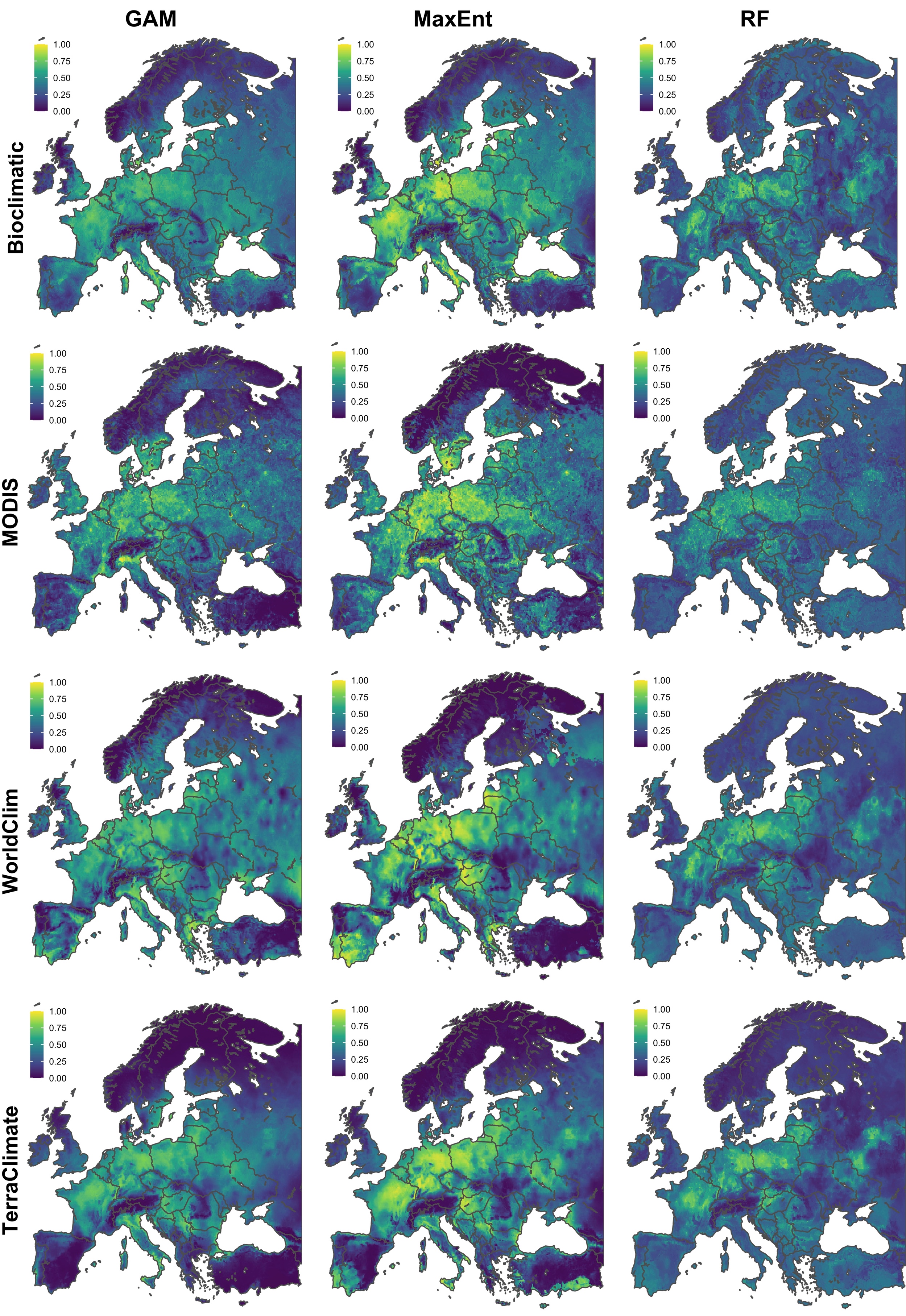


**Figure S14.** The predicted environmental suitability for *Dermacentor reticulatus* in Europe using different modelling approaches, including three modelling algorithms [random forests (RF), maximum entropy (MaxEnt) and generalised additive models (GAM)] and four explanatory variable sets (bioclimatic variables, WorldClim, TerraClimate and MODIS satellite-derived variables) using a 200km buffer training extent.


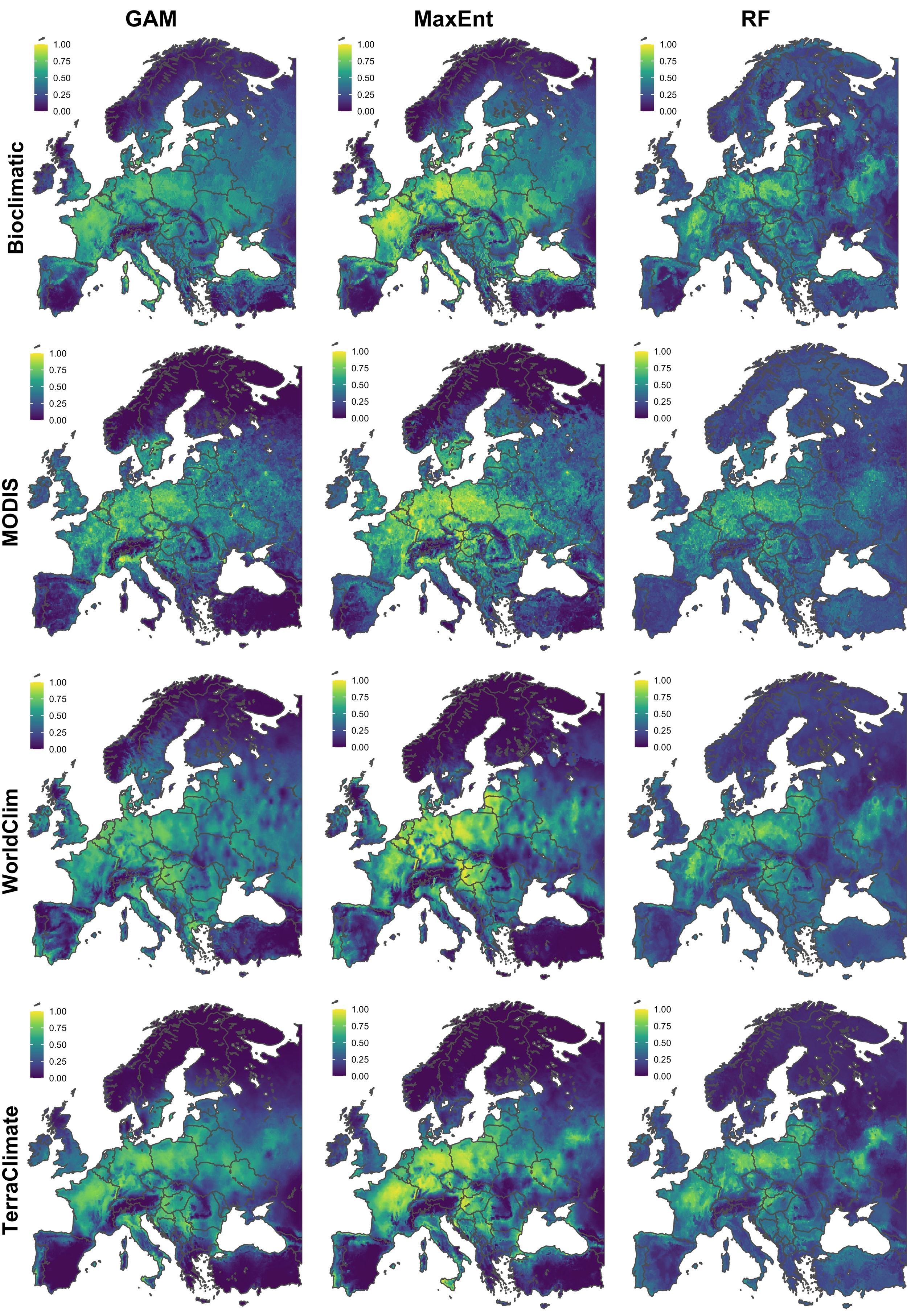


**Figure S15.** The predicted environmental suitability for *Dermacentor reticulatus* in Europe using different modelling approaches, including three modelling algorithms [random forests (RF), maximum entropy (MaxEnt) and generalised additive models (GAM)] and four explanatory variable sets (bioclimatic variables, WorldClim, TerraClimate and MODIS satellite-derived variables) using a 300km buffer training extent.


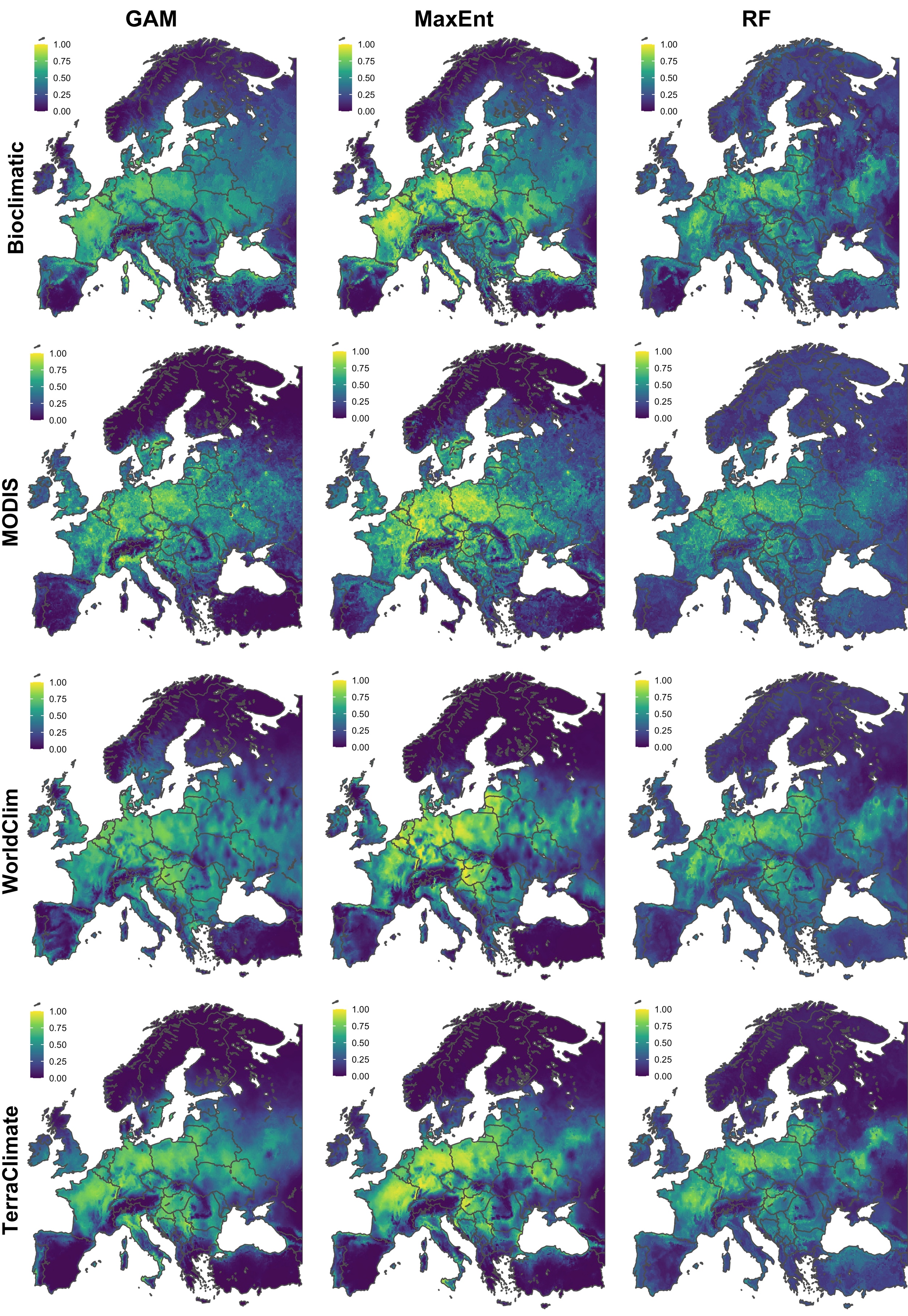


**Figure S16.** The predicted environmental suitability for *Dermacentor reticulatus* in Europe using different modelling approaches, including three modelling algorithms [random forests (RF), maximum entropy (MaxEnt) and generalised additive models (GAM)] and four explanatory variable sets (bioclimatic variables, WorldClim, TerraClimate and MODIS satellite-derived variables) using a 400km buffer training extent.


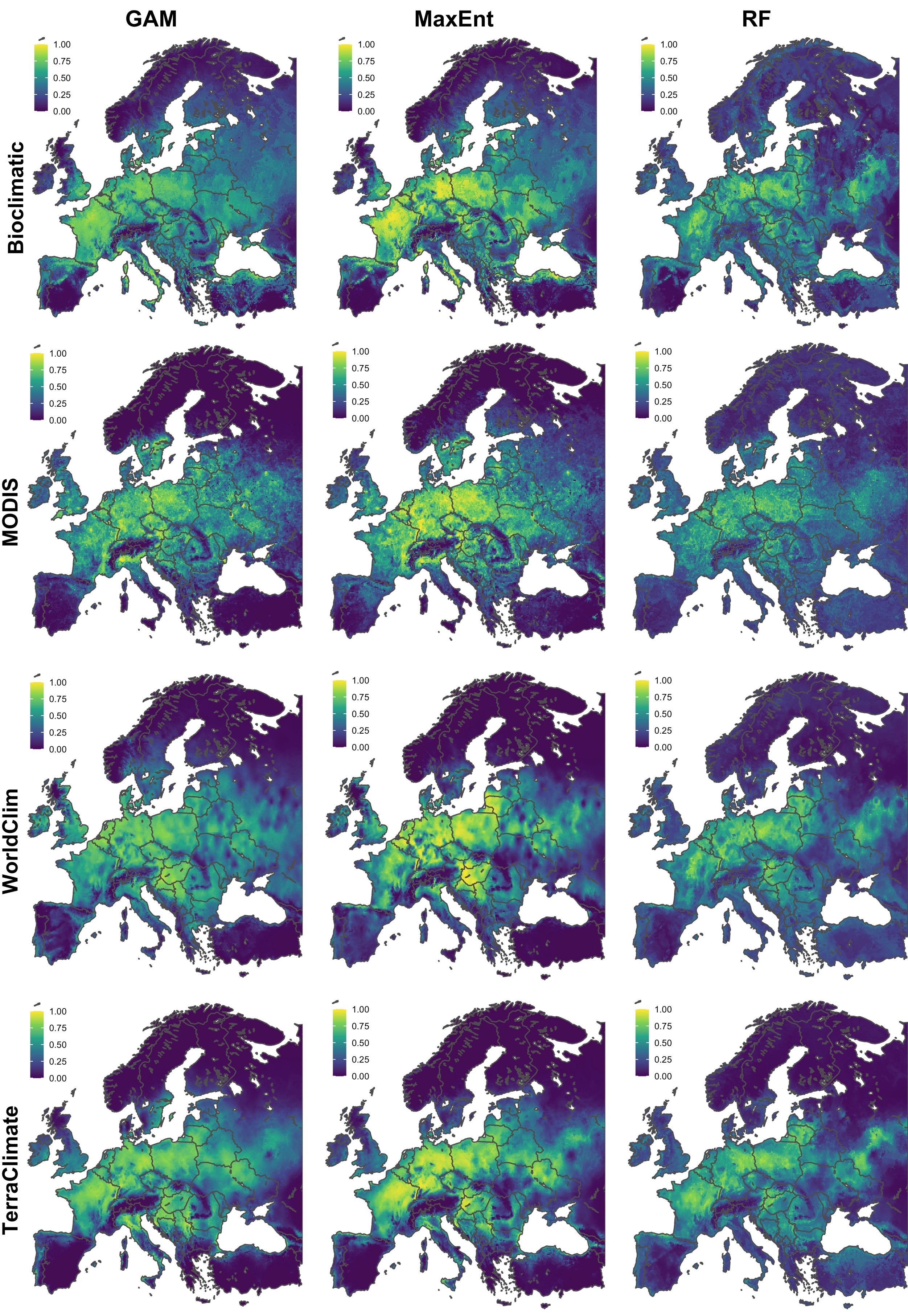


**Figure S17.** The predicted environmental suitability for *Dermacentor reticulatus* in Europe using different modelling approaches, including three modelling algorithms [random forests (RF), maximum entropy (MaxEnt) and generalised additive models (GAM)] and four explanatory variable sets (bioclimatic variables, WorldClim, TerraClimate and MODIS satellite-derived variables) using a 500km buffer training extent.


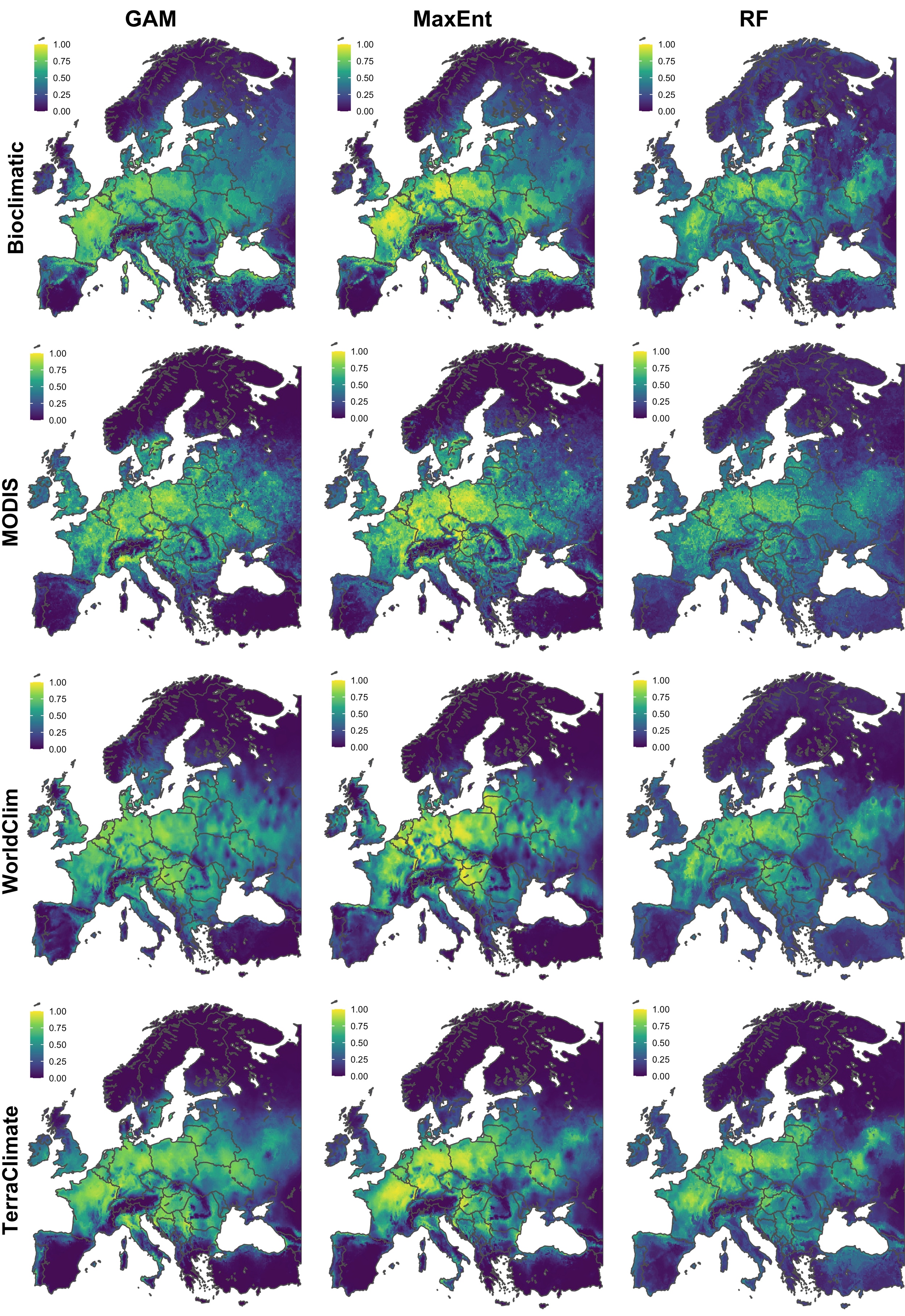


**Figure S18.** The predicted environmental suitability for *Dermacentor reticulatus* in Europe using different modelling approaches, including three modelling algorithms [random forests (RF), maximum entropy (MaxEnt) and generalised additive models (GAM)] and four explanatory variable sets (bioclimatic variables, WorldClim, TerraClimate and MODIS satellite-derived variables) using a 600km buffer training extent.


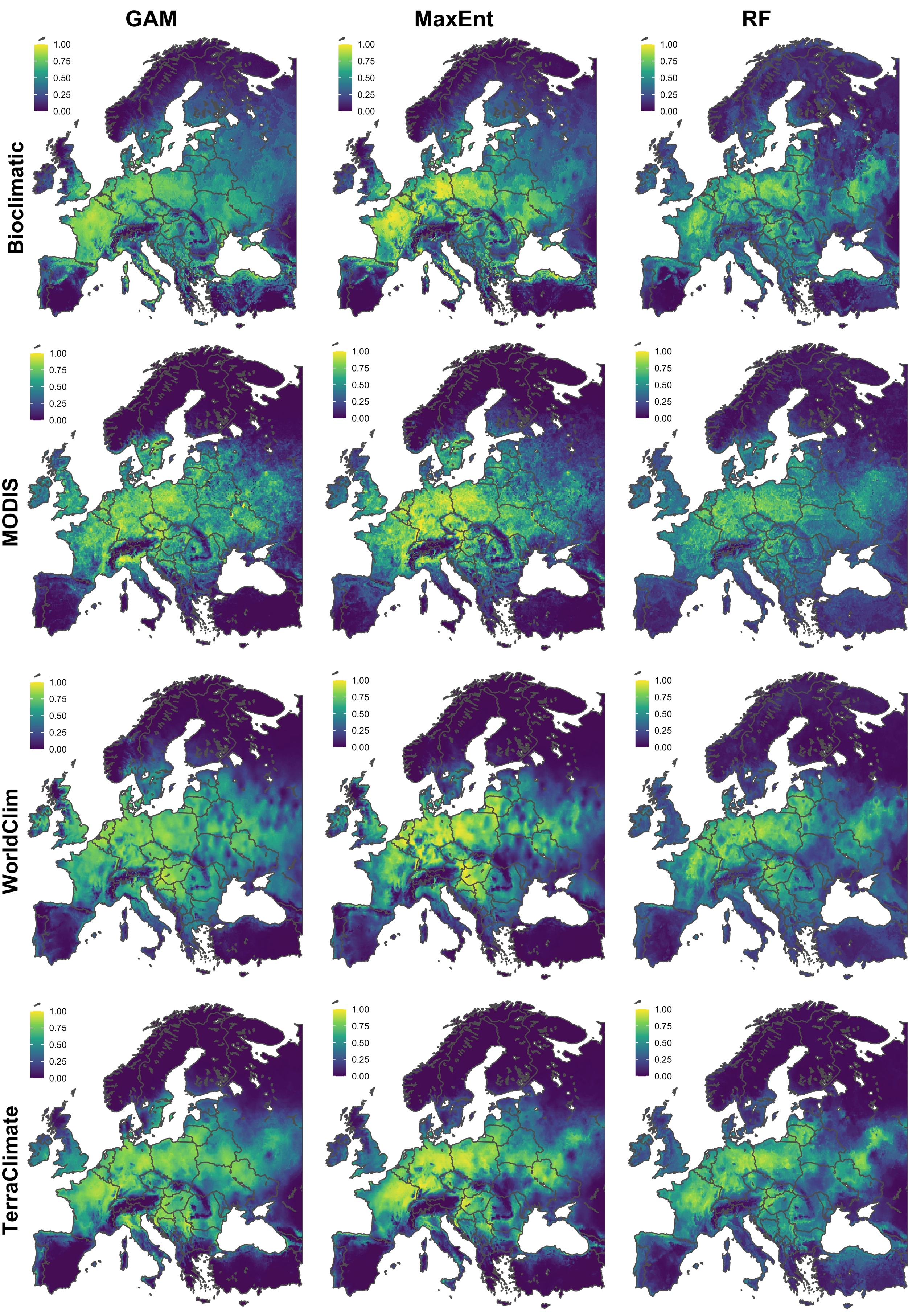


**Figure S19.** The predicted environmental suitability for *Dermacentor reticulatus* in Europe using different modelling approaches, including three modelling algorithms [random forests (RF), maximum entropy (MaxEnt) and generalised additive models (GAM)] and four explanatory variable sets (bioclimatic variables, WorldClim, TerraClimate and MODIS satellite-derived variables) using a 700km buffer training extent.


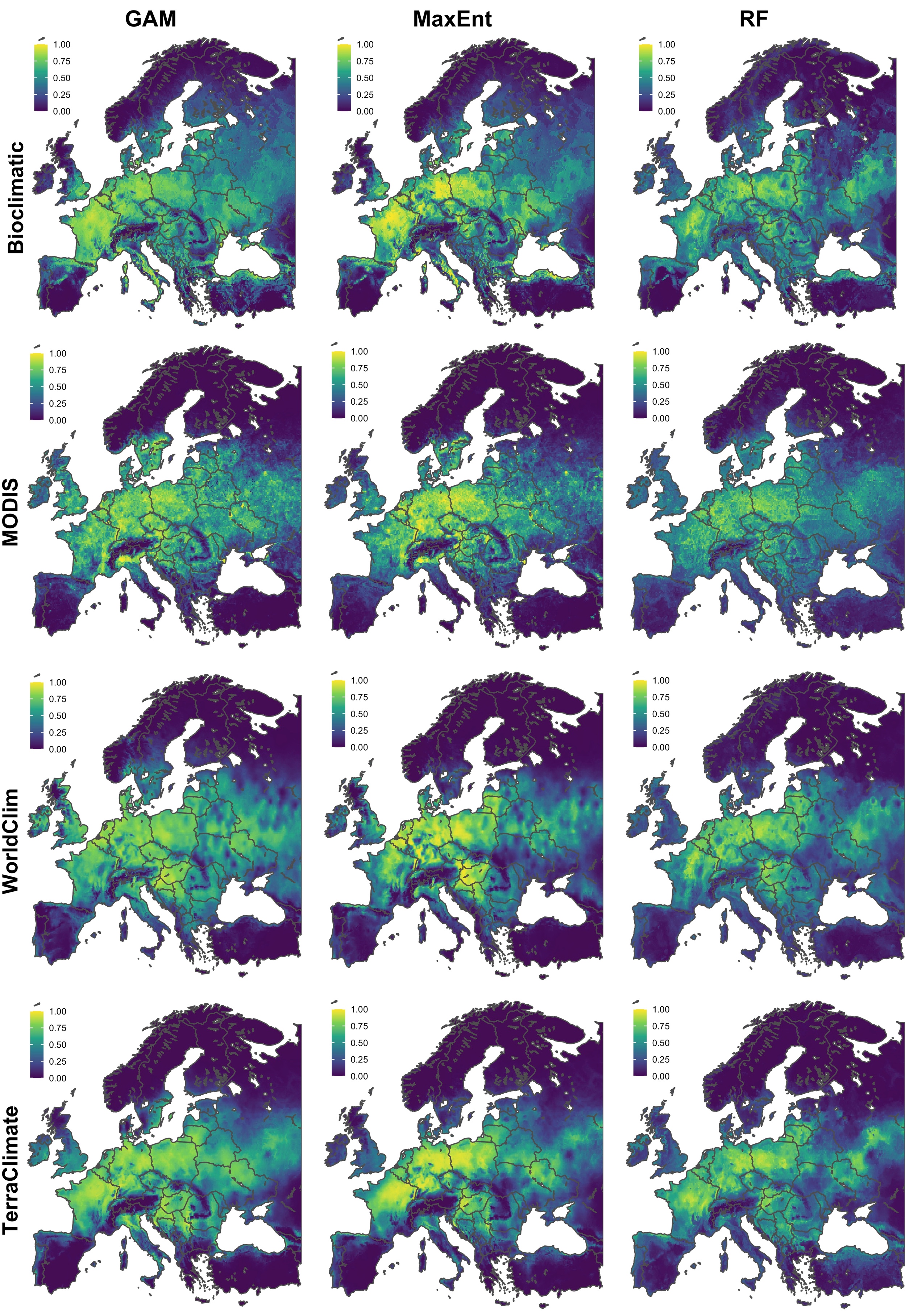


**Figure S20.** The predicted environmental suitability for *Dermacentor reticulatus* in Europe using different modelling approaches, including three modelling algorithms [random forests (RF), maximum entropy (MaxEnt) and generalised additive models (GAM)] and four explanatory variable sets (bioclimatic variables, WorldClim, TerraClimate and MODIS satellite-derived variables) using a training extent the size of Europe.
