# Supplementary material for: Predicting the distribution of Ixodes ricinus and Dermacentor reticulatus in Europe: a comparison of climate niche modelling approaches
Source: Parasit Vectors. 2023 Oct 25;16:384. doi: 10.1186/s13071-023-05959-y (PMC10601327; doi:10.1186/s13071-023-05959-y)
Supplement: Supplementary file 9 — Additional file 9: Text S1. The ODMAP (overview, data, model, assessment, prediction) protocol for reporting species distribution modelling 90. [file 13071_2023_5959_MOESM9_ESM.docx]

Predicting the distribution of *Ixodes ricinus* and *Dermacentor reticulatus* in Europe: a comparison of climate niche modelling approaches

– ODMAP Protocol –

Madeleine Noll, Richard Wall, Benjamin L. Makepeace, Hannah Newbury, Lukasz Adaszek, René Bødker, Agustín Estrada-Peña, Jacques Guillot, Isabel Pereira da Fonseca, Julia Probst, Paul Overgaauw, Christina Strube, Fathiah Zakham, Stefania Zanet, Hannah Rose Vineer,

2023-02-07

## Overview

#### Authorship

Contact : [madeleine.noll@liverpool.ac.uk](mailto:madeleine.noll@liverpool.ac.uk)

#### Model objective

Model objective: Mapping and interpolation

Target output: Continuous habitat suitability values

#### Focal Taxon

Focal Taxon: Three species of Ixodidae (hard ticks)

#### Location

Location: Europe

#### Scale of Analysis

Spatial extent: 11, 45, 34, 72 (xmin, xmax, ymin, ymax)

Spatial resolution: 10 x 10 km

Temporal extent: 1970-2021

Temporal resolution: 51 years

Boundary: political, natural

#### Biodiversity data

Observation type: systematic literature review, citizen science, scientific literature

Response data type: presence-only

#### Predictors

Predictor types: climatic

#### Hypotheses

Hypotheses: The species occurrence is related to specific climatic conditions.

#### Assumptions

Model assumptions:

Species: The species is in or has reached equilibrium with the study extent.

Occurrence Data: The cleaned and rarefied occurrence data comprise a dataset in which the entire study area has been randomly sampled, and the occurrence data represents the realised distribution of the species of interest.

Climate data: The climatic variables adequately represent the area occupied by the species of interest and are relevant to the species of interest’s climatic niche.

#### Algorithms

Modelling techniques: MaxEnt, Random Forest, Generalised Additive Models

Model complexity: Models should aim to capture the complex relationship between the species of interest occurrence data and climatic variables.

#### Workflow

Model workflow:

1) Combine species occurrence data from multiple sources.
2) Get individual species occurrence data and inspect and clean this data.
3) Remove spatial bias from the occurrence data.
4) Get the explanatory variables and project to the correct CRS and resolution.
5) Select the explanatory variables to use (not correlated).
6) Partition the data for cross-validation
7) Build and run the models
8) Evaluate the model by cross-validation and independent dataset.
9) Statistical comparison of model performance.
10) Graphical representations of model output and expert opinion.

#### Software

Software: R version R 4.1.2.

Packages: CoordinateCleaner (v.2.0-20), spThin (v.0.2.0) dismo (v.1.3-5), usdm (v.1.1-1.8) randomForest(v4.6-14), modEvA (v.3.0), blockCV (v.2.1.5), mgcv (v.1.8-38).

Code availability: On request of corresponding author

Data availability: All of the occurrence data is freely available with sources cited in the main text. A complete dataset is provided in supplementary material. The explanatory variables are also freely available and are available from a repository.

## Data

#### Biodiversity data

Taxon names: *Ixodes ricinus,* *Dermacentor reticulatus*

Ecological level: species

Data sources: Global Biodiversity Information Facility data (GBIF; GBIF.org; 4th February 2022; <https://doi.org/10.15468/dl.v7empg>). Estrada-Peña and de la Fuente (<https://doi.org/10.1038/sdata.2016.56>). Systematic Literature Review (Noll et al., 2023 In Review). All data cited in main text and available in supplementary material.

Sampling design: Not applicable. Secondary data used and therefore a collection of different sampling designs from different sources.

Sample size: 638 locations for *I. ricinus* (optimal thinning; 50 km, NNI = 0.996, Z = -0.176) and 153 for *D. reticulatus* (optimal thinning; 60 km, NNI = 1.014, Z = 0.337).

Clipping: Europe

Scaling: All analysis was conducted on a 10 x 10km grid to ensure the species occurrence data matched the resolution of the climate data. Occurrence point with uncertainty greater than 10km were excluded from the study.

Cleaning: Data were cleaned using the CoordinateClearner package in R to exclude points that were (i) missing or errors in coordinates, (ii) duplicated, (iii) coordinates fell within 1000m of country/province centroids, institutions, or capital cities, (iv) coordinates outside of the area of interest.

Absence data: No absence data were available.

Background data: 10,000 random background points were generated matching the extents of the occurrence data.

Errors and biases: To avoid the effects of sampling bias, the data were randomly spatially rarefied until the nearest neighbour index was close to 1, a random distribution.

#### Data partitioning

Training data: The data was selected over Europe which is well surveyed.

Validation data: The data was split into training and validation sets by spatial block cross-validation.

Test data: An independent tick occurrence dataset was acquired through a Pan-European tick surveillance project led by regional MSD Animal Health divisions. Veterinary practices across Europe were asked to submit ticks found on pets and record their geographic location (for details, see other papers in this volume). This independent dataset was thinned to such that no points were closer than 30km and resulted in 570 occurrence points for I. ricinus and 133 for D. reticulatus.

#### Predictor variables

Predictor variables: Four sets of climatic variables were used: BioClim, WorldClim, MODIS satellite derived data and TerraClimate data.

Data sources: Sources are cited in the main text.

Spatial extent: 11, 45, 34, 71 (xmin, xmax, ymin, ymax)

Spatial resolution: ~5 x 5 km

Coordinate reference system: WGS84

Temporal extent: BioClim (1970-2000), WorldClim (1970-2020), MODIS satellite derived data (2000-2000) and TerraClimate data (1970-2021).

Data processing: All variables were cropped and masked to Europe, and set to a resolution of 10x10 km to match occurrence data.

WorldClim, MODIS satellite derived data and TerraClimate data were subject to a Fourier transformation.

Dimension reduction: Variables were selected if they were ecologically relevant and had a variance inflation factor (VIF) below 10.

## Model

#### Variable pre-selection

Variable pre-selection: Variables were selected if they were ecologically relevant.

#### Multicollinearity

Multicollinearity: All variables had a variance inflation factor (VIF) below 10.

#### Model settings

MaxEnt: featureSet (Automatic), regularizationRule (1)

Random Forest: ntree (1000)

GAM: family (binomial), smoothTerms (s), Kvalue (dependent on the minimum number of unique values in explanatory variable).

#### Model selection - model averaging - ensembles

Model selection: Variables were chosen based on previous work and their ecological meaningfulness.

Model averaging: The average of each of the folds was determined.

#### Threshold selection

Threshold selection: Binary results were generated using a threshold which optimised the TSS of the model.

## Assessment

#### Performance statistics

Performance on training data: Area Under ROC Curve, True Skill Statistics, Miller’s Calibration Slope, Continuous Boyce Index, Omission Rate, Uncertainty Index

Performance on validation data: Area Under ROC Curve, True Skill Statistics, Miller’s Calibration Slope, Continuous Boyce Index, Omission Rate, Uncertainty Index

Performance on test data: Area Under ROC Curve, True Skill Statistics, Miller’s Calibration Slope, Continuous Boyce Index, Omission Rate, Uncertainty Index

#### Plausibility check

Expert judgement: All authors critically analysed the resultant maps.

## Prediction

#### Prediction output

Prediction unit: Habitat suitability 0-1

#### Uncertainty quantification

Algorithmic uncertainty: An uncertainty index was generated by finding the range between the minimum and maximum value of predicted environmental suitability for each cell within the five replicates for each modelling combination. This was to show the uncertainty in the results derived from different subsets of occurrence data. The sum of the uncertainty was then normalised between 0-1 to allow for comparison.
